# Synthesis and Anticancer Activity Evaluation of 5-[2-Chloro-3-(4-nitrophenyl)-2-propenylidene]-4-thiazolidinones

**DOI:** 10.3390/molecules26103057

**Published:** 2021-05-20

**Authors:** Kamila Buzun, Anna Kryshchyshyn-Dylevych, Julia Senkiv, Olexandra Roman, Andrzej Gzella, Krzysztof Bielawski, Anna Bielawska, Roman Lesyk

**Affiliations:** 1 Department of Biotechnology, Medical University of Bialystok, Jana Kilińskiego 1, 15-089 Bialystok, Poland; kamila.buzun@umb.edu.pl (K.B.); anna.bielawska@umb.edu.pl (A.B.); 2 Department of Pharmaceutical, Organic and Bioorganic Chemistry, Danylo Halytsky Lviv National Medical University, Pekarska 69, 79010 Lviv, Ukraine; kryshchyshyn.a@gmail.com (A.K.-D.); lesia_roman@ukr.net (O.R.); 3 Institute of Cell Biology of National Academy of Sciences of Ukraine, 14/16 Drahomanov Str., 79005 Lviv, Ukraine; yu.senkiv@gmail.com; 4 Department of Organic Chemistry, Poznan University of Medical Sciences, Grunwaldzka 6, 60-780 Poznan, Poland; akgzella@ump.edu.pl; 5 Department of Synthesis and Technology of Drugs, Medical University of Bialystok, 15-089 Bialystok, Poland; kbiel@umb.edu.pl; 6 Department of Public Health, Dietetics and Lifestyle Disorders, Faculty of Medicine, University of Information Technology and Management in Rzeszow, 35-225 Rzeszow, Poland

**Keywords:** synthesis, 4-thiazolidinones, Ciminalum, anticancer activity, SAR analysis

## Abstract

A series of novel 5-[(*Z*,2*Z*)-2-chloro-3-(4-nitrophenyl)-2-propenylidene]-thiazolidinones (Ciminalum–thiazolidinone hybrid molecules) have been synthesized. Anticancer activity screening toward the NCI60 cell lines panel, gastric cancer (AGS), human colon cancer (DLD-1), and breast cancer (MCF-7 and MDA-MB-231) cell lines allowed the identification of 3-{5-[(*Z*,2*Z*)-2-chloro-3-(4-nitrophenyl)-2-propenylidene]-4-oxo-2-thioxothiazolidin-3-yl}propanoic acid (**2h**) with the highest level of antimitotic activity with mean GI_50_/TGI values of 1.57/13.3 μM and a certain sensitivity profile against leukemia (MOLT-4, SR), colon cancer (SW-620), CNS cancer (SF-539), melanoma (SK-MEL-5), gastric cancer (AGS), human colon cancer (DLD-1), and breast cancers (MCF-7 and MDA-MB-231) cell lines. The hit compounds **2f**, **2i**, **2j**, and **2h** have been found to have low toxicity toward normal human blood lymphocytes and a fairly wide therapeutic range. The significant role of the 2-chloro-3-(4-nitrophenyl)prop-2-enylidene (*Ciminalum*) substituent in the 5 position and the substituent’s nature in the position 3 of core heterocycle in the anticancer cytotoxicity levels of 4-thiazolidinone derivatives have been established

## 1. Introduction

In recent years, one of successful directions in the structure design of “drug-like” molecules is the “hybrid-pharmacophore” approach that involves combining different fragments in one molecule that can be parts, biomimetics, and/or bioisosteres of biologically active molecules or drugs. This strategy allows potentiating the desired action or appearance of new effects [1,2,3] and can be relevant for the search for new highly active compounds based on 4-thiazolidinones as effective biophores. Thus, modern studies of the pharmacological potential of thiazolidinones have significantly expanded the range of their activity, including anticancer, antibacterial, antifungal, antiviral, antiparasitic, and anti-tuberculosis. Along with this, there is indisputable evidence of the affinity of these derivatives for biotargets involved in the biochemical processes of tumor cell growth (TNF-α-TNFRc-1, JSP-1, antiapoptotic complex Bcl-XL-BH3), the microorganisms life cycle (UDP-NMurNA/L-Ala-ligase), the development of inflammatory conditions (COX-2/5-LOX), and the development of type II diabetes mellitus (PPARγ) [4,5].

**Figure 1:**
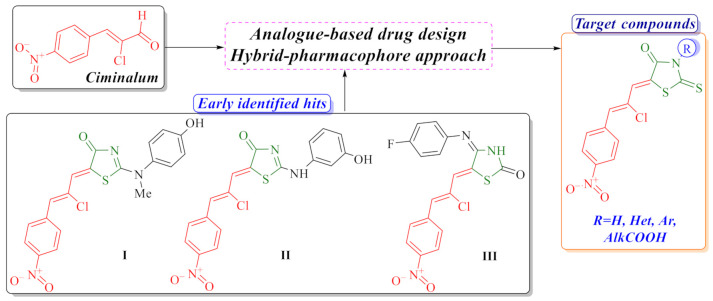
Background of the target compounds design.

Based on our previous research, we have established that the combination of a thiazolidinone moiety and a structural fragment of the *Ciminalum* in one hybrid molecule is an effective approach for the design of potential anticancer agents [6,7]. *Ciminalum* (*p*-nitro-α-chlorocinnamic aldehyde or (2*Z*)-2-chloro-3-(4-nitrophenyl)prop-2-enal, CAS 3626-97-9) is an active antimicrobial agent against Gram-positive and Gram-negative microorganisms. *Ciminalum* was used as a drug in medical practice in the former Soviet Union (Figure 1) [8]. *Ciminalum*–thiazolidinone hybrid molecules (namely 5-[(*Z*,2*Z*)-2-chloro-3-(4-nitrophenyl)-2-propenylidene]-4-thiazolidinones) showed a significant cytotoxic effect on tumor cells. It is important to note that the presence of a *Ciminalum* moiety in position 5 of the thiazolidinone ring is key to the manifestation of biological activity. Thus, early hits **I** and **II** (Figure 1) possessed a selectively high effect on leukemia, melanoma, lung, colon, CNS, ovarian, renal, prostate, and breast cancers cell lines at micro- and submicromolar levels that is probably associated with the immunosuppressive activity [7]. Early anticancer hit **III** has induced and activated specific signaling apoptotic pathways in tumor cells [6]. Thus, compound **III** leads to weak caspase-7 activation and a weak cleavage of PARP-1 and DFF45 in the Jurkat T-cells. However, this *Ciminalum*-thiazolidinone hybrid may be involved in the caspase-independent, AIF-mediated apoptosis. AIF (apoptosis-inducing factor) is known to induce the mitochondria-mediated caspase-independent apoptosis. Derivative **III** leads to the activation of intrinsic apoptotic pathways, mediated by mitochondria, and caspases seem to play a minor role here.

Our research was aimed at optimization of anticancer activity profile of 5-[(*Z*,2*Z*)-2-chloro-3-(4-nitrophenyl)-2-propenylidene]-4-thiazolidinones and SAR analysis within these series in accordance with our systematic study of anticancer activity of thiazolidinone-related derivatives [9,10].

## 2. Results and Discussion

### 2.1. Chemistry

**Figure 2:**
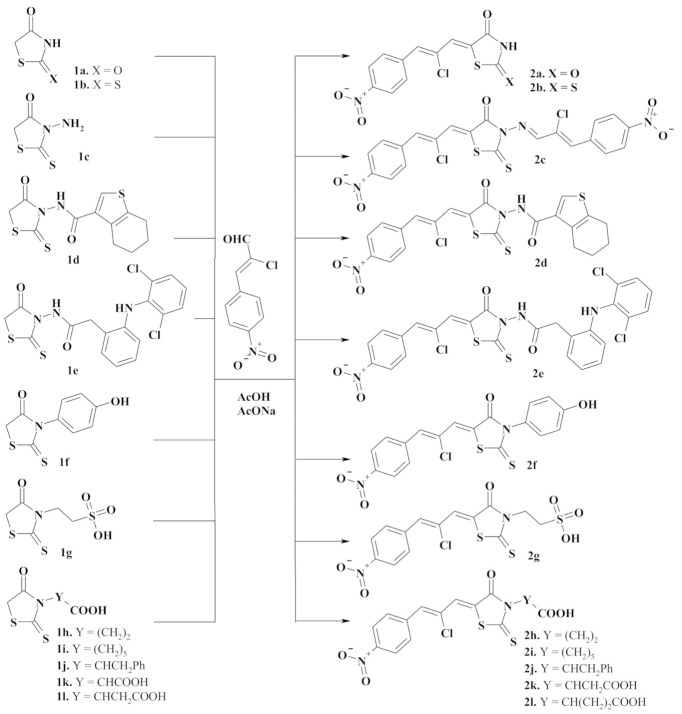
Synthesis of 5-[(*Z*,2*Z*)-2-chloro-3-(4-nitrophenyl)-2-propenylidene]-4-thiazolidinones **2a-m**. Reagents and conditions: appropriate 4-thiazolidinone **1a-l** (0.01 mol), (2*Z*)-2-chloro-3-(4-nitrophenyl)prop-2-enal (0.010 mol, in the case of 3-aminorhodanine **1e** 0.02 mol), AcONa (0.01 mol), AcOH (20 mL), reflux, 3 h, 68–83%.

The synthetic approach to target compounds design was based on 4-thiazolidinone derivatives as active methylene heterocycles in Knoevenagel reaction with (2*Z*)-2-chloro-3-(4-nitrophenyl)prop-2-enal (*Ciminalum*) as an oxo-compound (Figure 2). 

The starting 2,4-thiazolidinedione **1a** and 2-thioxo-4-thiazolidinone (rhodanine) derivatives **1b-l** were obtained according to described procedures. We used three synthetic approaches to this end: (1) [2+3]-cyclocondensation of chloroacetic acid with thiourea (**1a** [11]) or ammonium thiocyanate (**1b** [12]); (2) dithiocarbamate method of 3-substituted 2-thioxo-4-thiazolidinone (rhodanine) derivatives synthesis (**1c** [13], **1g–l** [14,15,16,17,18,19]); (3) the reaction of trithiocarbonyl diglycolic acid with amino compounds (**1d–f** [20,21]). 

Target *Ciminalum*-thiazolidinone hybrid molecules **2a–l** were synthesized via the Knoevenagel condensation of (2*Z*)-2-chloro-3-(4-nitrophenyl)prop-2-enal and appropriate 4-thiazolidinone in the presence of sodium acetate under reflux in acetic acid (Figure 2). In the case of 3-aminorhodanine **1c**, in parallel with the Knoevenagel condensation, the reaction with amino group and formation of appropriate azomethine derivative **2c** was observed. The data characterizing synthesized novel 4-thiazolidinones are presented in the experimental part. Analytical and spectral data (^1^H and ^13^C-NMR, LCMS) confirmed the structure of the synthesized compounds. The ^1^H-NMR spectra of the synthesized compounds are characterized by the signals of the *Cyminalum* residue in the form of two singlets at 7.55–8.01 and 7.88–8.07 ppm for the CH=CCl-CH= group as well as two doublets of the *p*-nitrophenyl substituent at 8.00 and 8.30 ppm. For compound **2g**, these signals were slightly shifted into a strong magnetic field and appeared as two singlets at 6.89 and 7.19 ppm and two doublets at 7.23 and 7.51 ppm. In the ^13^C-NMR spectra of rhodanine derivatives signals of C=O and C=S groups of the core heterocycle were characteristic and appeared at 158.7–174.4 and 193.5–199.3 ppm, respectively.

**Figure 3:**
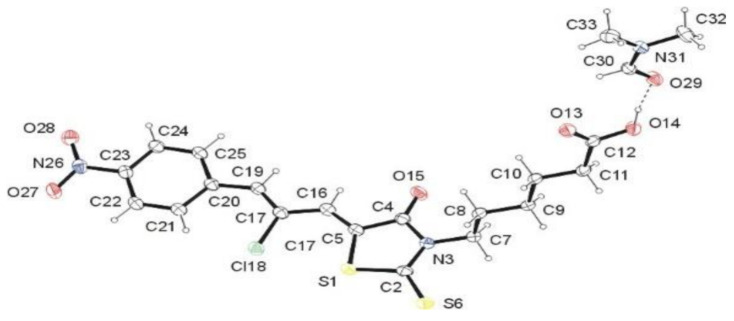
ORTEP view of **2i**·DMF showing the atomic labeling scheme. Non-H atoms are drawn as 30% probability displacement ellipsoids and H atoms are drawn as spheres of an arbitrary radius.

Structural features of the synthesized *Ciminalum*–thiazolidinone hybrid molecules were confirmed by single-crystal X-ray diffraction study of compound **2i**. As follows from the X-ray analysis, the investigated compound has the structure of 6-{5-[(*Z*,2*Z*)-2-chloro-3-(4-nitrophenyl)-2-propenylidene]-4-oxo-2-thioxothiazolidin-3-yl}hexanoic acid (**2i**) and crystallizes as dimethylformamide solvate in a molar ratio of 1:1 (Figure 3). 5-Carboxypentyl group located at N-3 atom adopts anticlinal conformation with respect to C2–N3 bond belonging to 2-thioxo-4-thiazolidinone moiety. This arrangement is confirmed by the torsion angle C2–N3–C7–C12 [−101.19(15)°]. The 3-(4-nitrophenyl)-2-chloroprop-2-en-1-ylidene residue at the C-5 atom assumes *Z* configuration with respect to the S1–C5 bond. The torsion angle S1–C5–C16–C17 has the value of 2.0(3)°. The system formed by the named residue and 2-thioxo-1,3-thiazolidin-4-one system is approximately planar [r.m.s.d. = 0.0949 Å].

The conformation of the molecule of **2i** is stabilized by the intermolecular hydrogen bonding. The hydrogen bonds C22–H···S6^iii^ and C25–H25···O29^iv^ (Table 1) stabilize the almost coplanar arrangement of the 3-(4-nitrophenyl)-2-chloroprop-2-en-1-ylidene and 2-thioxo-4-thiazolidinone moieties. Moreover, the hydrogen bonds O14–H14···O29, C11–H11A···O13i, and C11–H11B···O15^ii^ (Table 1) stabilize the spatial arrangement of the 5-carboxypentyl group. The C5═C16 [1.345(2) Å] and C17═C19 [1.351(2) Å] bond lengths confirmed the occurrence of a double bonds between these carbon atoms (Figure 3).

### 2.2. In Vitro Evaluation of the Anticancer Activity

At the first stage of biological activity study, the antitumor activity screening of the selected compounds **2b**, **2c**, **2f**, **2h**, and **2j** was performed according to the NCI DTP (USA) standard protocol at the concentrations ranging from 10^−4^ to 10^−8^ M toward 60 tumor cell lines [22,23,24,25]. The percentage of growth was evaluated spectrophotometrically versus controls not treated with test agents after 48 h exposure and using SRB protein assay to estimate cell viability or growth. Dose–response parameters were calculated for each cell line: GI_50_—molar concentration of the compound that inhibits 50% net cell growth; TGI—molar concentration of the compound leading to the total inhibition; and LC_50_—molar concentration of the compound leading to 50% net cell death. Furthermore, mean graph midpoints (MG_MID) were calculated for each of the parameters, giving an average activity parameter over all cell lines for the tested compound. For the MG_MID calculation, insensitive cell lines were included with the highest concentration tested.

The obtained results of screening evaluation of *Ciminalum*–thiazolidinone hybrids confirmed their significant anticancer activity (Table 2). Thus, compounds **2f** and **2h** inhibited the growth of all tested cancer cell lines at submicromolar and micromolar concentrations. The average meanings of three dose–response parameters GI_50_, TGI, and LC_50_ were 2.80/32.3/80.8 µM (**2f**) and 1.57/13.3/65.0 µM (**2h**), respectively. It is important to note that the most active compound **2h** was active in the GI_50_ concentration range of < 0.01–0.02 μM toward the following cell lines: MOLT-4, SR (Leukemia); SW-620 (Colon cancer); SF-539 (CNS cancer); SK-MEL-5 (Melanoma). Regarding the preliminary SAR analysis, it is worth mentioning that the presence of the (*Z*,2*Z*)-2-chloro-3-(4-nitrophenyl)-2-propenylidene moiety turned out to be a necessary requirement for achieving the anticancer effects. Moreover, the substituent nature at position 3 of the 4-thiazolidinone ring is important. Derivatives with carboxylic acids residues (**2h**, **2j**) and *p*-hydroxyphenyl substituent (**2f**) proved to be the most effective. The absence of a substituent in position 3 (**2b**) or an additional fragment of the *Cyminalum* (**2c**) leads to the weakening of anticancer cytotoxicity.

The selectivity index (SI) obtained by dividing the full panel MG-MID (mM) of the tested compound by their individual subpanel MG-MID (mM) was considered as a measure of selectivity of anticancer activity (Table 3). Ratios between 3 and 6 mean moderate selectivity, ratios greater than 6 indicate high selectivity toward the corresponding cell line, while compounds not meeting either of these criteria are rated nonselective [26]. The most active compounds **2f** and **2h** in the present study were found to be high selective toward the leukemia subpanel at GI_50_ levels (selectivity indices 9.89 and 10.73, respectively). Compound **2j** possessed high selectivity toward the CNS cancer subpanel at both the TGI and LC_50_ levels (selectivity index 11.53 and 10.25, respectively). In general, it is worth noting the selectivity of action against leukemia cell lines for the studied class of heterocyclic compounds.

In the second stage of the research, *Ciminalum*-thiazolidinone hybrids were investigated for antitumor activity on the lines of gastric cancer (AGS), human colon cancer (DLD-1), and breast cancers (MCF-7 and MDA-MB-231). The study was performed in the MTT assay according to the method described previously [27]. The studied cancer line was sensitive to the action of the studied compounds that inhibited its growth in micromolar ranges of GI_50_. The hit compounds that inhibited the growth of all four cancer lines with the lowest GI_50_ values were [5-[2-chloro-3-(4-nitrophenyl)prop-2-enylidene]-rhodanines **2c**, **2d**, **2h**, and **2i** (Table 4). Moreover, it is important to note the high cytotoxic effect of rhodanine-3-carboxylic acid derivatives **2h** and **2i** toward breast cancer lines MCF-7 and MDA-MB-231 at the GI_50_ level of 0.95–1.74 μM, which is consistent with previous data obtained according to DTP NCI protocol (Table 2).

**Figure 4:**
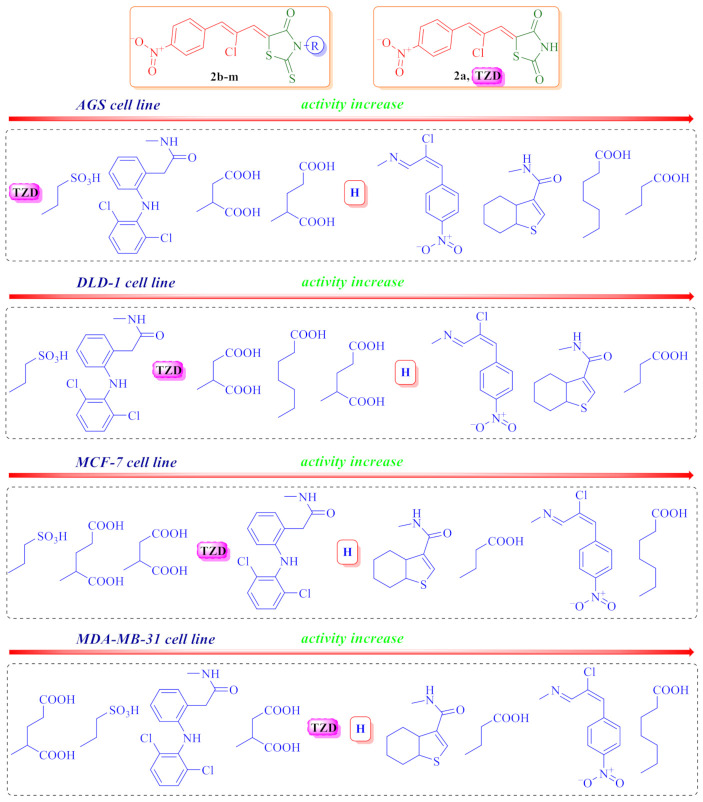
Impact of different substituents in N3 position of the rhodanine core on the anticancer activity levels.

Regarding the SAR analysis (Figure 4), the significant role of the 5-[2-chloro-3-(4-nitrophenyl)prop-2-enylidene (*Ciminalum*) substituent in the anticancer cytotoxicity appearance was confirmed. Moreover, the presence of a thioxo group in position 2 of the core heterocycle is more important than the oxo group, as evidenced by the lower activity of the thiazolidinedione **2b** compared to a structurally close rhodanine derivative **2a**. The role of the substituents nature in position 3 of the rhodanine core on the level of anticancer cytotoxicity level is interesting and important for further in-depth research and the design of drug-like molecules. Thus, the most effective is the presence of carboxylic acids residues, among which fragments of propanoic (**2h**) and hexanoic (**2i**) acids are considered to be important for cytotoxicity toward AGS, DLD-1, MCF-7, and MDA-MB-231 cell lines. The introduction of an additional carboxylic group reduced the effect of derivatives by about 10 times (compounds **2k** and **2l**). Replacing the carboxyl group with a sulfo group had reduced the activity more significantly (compound **2g**). In addition to the carboxylic acid residues, an additional *Ciminalum* fragment (**2c**) or 4,5,6,7-tetrahydrobenzo[*b*]thiophen-3-ylcarboxamide moiety (**2d**) at position 3 of the rhodanine cycle were also important for the anticancer activity.

**Figure 5:**
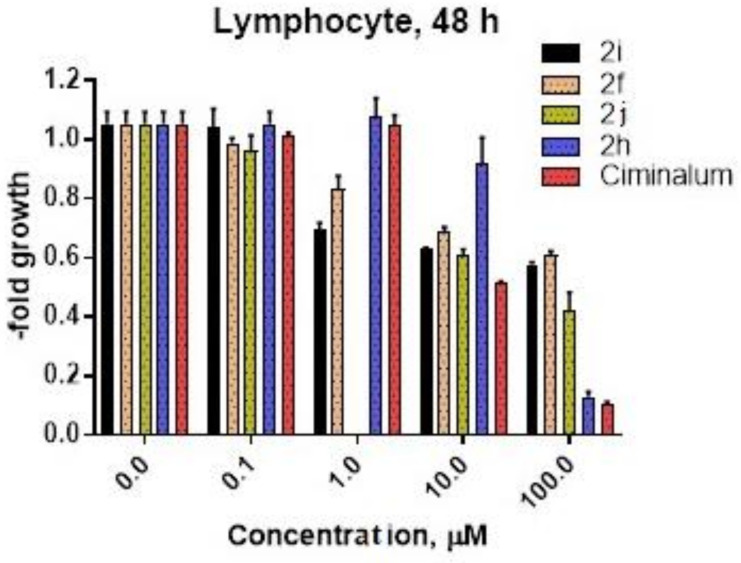
Human lymphocyte viability after 48 h of **2f**, **2i**, **2j**, **2h**, and *Ciminalum* drug exposure was estimated by MTT assay.

Another part of our study was to determine the influence of compounds **2f**, **2i**, **2j**, and **2h** on normal human blood lymphocytes (Figure 5). GI_50_ values for compounds **2j** and **2h** were 48.97 μM and 54.54 μM correspondingly. Compounds **2i** and **2f** do not reach GI_50_ up to 100 μM after 48 h incubation. The pure *Ciminalum* has the lowest IC_50_ value (GI_50_ = 10.4 μM) for human normal lymphocytes. Thus, normal blood lymphocytes are blood cells, as well as cells of leukemia cell lines, therapeutic index (TI) of compounds **2f**, **2h**, and **2j** was calculated as GI_50_ (normal blood lymphocyte)/GI_50_ (leukemia cell line) (Table 5).

## 3. Materials and Methods

### 3.1. General Information

All reagents and solvents were purchased from commercial suppliers and were used directly without further purification. NMR spectra were determined with Varian Unity Plus 400 (400 MHz) and Bruker 170 Avance 500 (500 MHz) spectrometers, in DMSO-*d*_6_ using tetramethylsilane (TMS) as an internal standard. Melting points were measured on a Kofler hot-stage and are uncorrected. LC-MS was performed using a system with an Agilent 1100 Series HPLC equipped with diode-array detector and Agilent LC\MSD SL mass-selective detector using chemical ionization at atmospheric pressure (APCI). The NMR and LCMS spectra of compounds 2a–l are presented in Figures S1–S32.

### 3.2. Synthesis of 5-[(Z,2Z)-2-chloro-3-(4-nitrophenyl)-2-propenylidene]-thiazolidinone derivatives (***2a-l***)

A mixture of (2Z)-2-chloro-3-(4-nitrophenyl)prop-2-enal (0.01 mol) and appropriate 4-thiazolidinone (0.01 mol) in the medium of acetic acid (20 mL) and the presence of sodium acetate (0.01 mol) was refluxed for 3 h. Obtained solid product was collected after cooling by filtration and recrystallized from the mixture DMF-ethanol (1:2).

5-[(*Z*,2*Z*)-2-Chloro-3-(4-nitrophenyl)-2-propenylidene]-2,4-thiazolidinedione (**2a**). Yield: 78%, mp >270 °C. ^1^H-NMR (400 MHz, DMSO-*d*_6_): δ (ppm) 7.70 (s, 1H, CH=), 7.88 (s, 1H, CH=), 8.00 (d, 2H, *J* = 7.5 Hz, arom.), 8.31 (d, 2H, *J* = 8.0 Hz, arom.), 12.70 (br.s, 1H, NH). LCMS (ESI): *m*/*z* 309.9/312.0 (95.58%, [M + H]^+^). Anal. Calc. for C_12_H_7_ClN_2_O_4_S: C 46.39%; H 2.27%; N 9.02%. Found: C 46.50%; H 2.40%; N 8.90%.

5-[(*Z*,2*Z*)-2-Chloro-3-(4-nitrophenyl)-2-propenylidene]-2-thioxo-4-thiazolidinone (**2b**). Yield: 81%, mp 251–253 °C. ^1^H-NMR (400 MHz, DMSO-*d*_6_): δ (ppm) 7.55 (s, 1H, CH=), 7.94 (s, 1H, CH=), 8.01 (d, 2H, *J* = 8.5 Hz, arom.), 8.31 (d, 2H, *J* = 8.2 Hz, arom.), 13.91 (br.s, 1H, NH). LCMS (ESI): *m*/*z* 324.9/326.9 (100%, [M + H]^+^). Anal. Calc. for C_12_H_7_ClN_2_O_3_S_2_: C 44.11%; H 2.16%; N 8.57%. Found: C 44.00%; H 2.25%; N 8.70%.

5-[(*Z*,2*Z*)-2-Chloro-3-(4-nitrophenyl)-2-propenylidene]-3-[(*Z*,2*Z*)-2-chloro-3-(4-nitrophenyl)-2-propenylideneamino]-2-thioxo-4-thiazolidinone (**2c**). Yield: 74%, mp >260 °C. ^1^H-NMR (400 MHz, DMSO-*d*_6_): δ (ppm) 7.79 (s, 1H, =CH), 8.01 (s, 1H, =CH), 8.06 (d, 2H, *J* = 8.1 Hz, arom.), 8.06 (s, 1H, =CH), 8.07 (s, 1H, =CH), 8.18 (d, 2H, *J* = 8.8 Hz, arom.), 8.33 (d, 2H, *J* = 8.8 Hz, arom.), 8.37 (d, 2H, *J* = 8.1 Hz, arom.), 8.98 (s, 1H, CH=N). LCMS (ESI): *m*/*z* 535.0 (95.05%, [M + H]^+^). Anal. Calc. for C_21_H_12_Cl_2_N_4_O_5_S_2_: C 47.11%; H 2.26%; N 10.46%. Found: C 47.00%; H 2.15%; N 10.65%.

5-[(*Z*,2*Z*)-2-Chloro-3-(4-nitrophenyl)-2-propenylidene]-4-oxo-3-(4,5,6,7-tetrahydrobenzo[b]thiophen-3-ylcarboxamido)-2-thioxo-4-thiazolidinone (**2d**). Yield: 80%, mp >230 °C. ^1^H-NMR (400 MHz, DMSO-*d*_6_): δ (ppm) 1.68–1.74 (m, 4H, 2*CH_2_), 2.67–2.78 (m, 4H, 2*CH_2_), 7.87 (s, 1H, s, 1H, CH=), 8.03–8.09 (m, 3H, arom., CH=), 8.10 (s, 1H, s, 1H, thiophene), 8.33 (d, 2H, *J* = 8.3 Hz, arom.), 11.46 (s, 1H, NH). ^13^C-NMR (100 MHz, DMSO-*d*_6_): δ (ppm) 27.2, 27.8, 29.8, 30.4, 128.2, 129.1, 134.8, 136.2, 137.9, 140.3, 142.5, 144.3, 144.8, 145.0, 152.7, 168.7 (C=O), 177.9 (C=O), 196.4 (C=S). LCMS (ESI): *m*/*z* 504.0/506.0 (100%, [M-H]^+^). Anal. Calc. for C_21_H_16_ClN_3_O_4_S_3_: C 49.85%; H 3.19%; N 8.30%. Found: C 50.00%; H 3.15%; N 8.35%.

*N^1^*-{5-[(*Z*,2*Z*)-2-Chloro-3-(4-nitrophenyl)-2-propenylidene]-4-oxo-2-thioxothiazolidin-3-yl}-2-[2-(2,6-dichloroanilino)phenyl]acetamide (**2e**). Yield: 74%, mp 257–258 °C. ^1^H-NMR (400 MHz, DMSO-*d*_6_): δ (ppm) 3.85 (d, 1H, *J* = 14.8 Hz, CH_2_), 3.90 (d, 1H, *J* = 14.8 Hz, CH_2_), 6.30 (d, 1H, *J* = 7.6 Hz, arom.), 7.08 (t, 1H, *J* = 7.5 Hz, arom.), 7.19 (t, 1H, *J* = 8.0 Hz, arom.) 7.29 (s, 1H, NH), 7.34 (d, 1H, *J* = 7.3 Hz, arom.), 7.53 (d, 2H, *J* = 8.0 Hz, arom.), 7.83 (s, 1H, =CH), 8.05 (d, 2H, *J* = 7.7 Hz, arom.), 8.06 (s, 1H, =CH), 8.33 (2H, arom., *J* = 8.4 Hz, arom.), 11.68 (s, 1H, NH). ^13^C-NMR (100 MHz, DMSO-*d*_6_): δ (ppm) 41.6 (CH_2_), 121.4, 121.5, 126.1, 126.2, 129.1, 129.2, 130.8, 132.9, 134.4, 134.8, 135.3, 135.6, 136.2, 137.7, 142.3, 144.8, 152.7, 168.3 (C=O), 177.0 (C=O), 195.9 (C=S). LCMS (ESI): *m*/*z* 618.8/621.6 (96.2%, [M - H]^+^). Anal. Calc. for C_27_H_16_Cl_3_N_4_O_4_S_2_: C 50.37%; H 2.76%; N 9.04%. Found: C 50.20%; H 2.85%; N 9.15%.

5-[(*Z*,2*Z*)-2-Chloro-3-(4-nitrophenyl)-2-propenylidene]-3-(4-hydroxyphenyl)-2-thioxo-4-thiazolidinone (**2f**). Yield: 76%, mp >260 °C. ^1^H-NMR (400 MHz, DMSO-*d*_6_): δ (ppm) 6.89 (d, 2H, *J* = 8.4 Hz, arom.), 7.16 (d, 2H, *J* = 8.4 Hz, arom.), 7.72 (s, 1H, =CH), 8.02 (s, 1H, =CH), 8.05 (d, 2H, *J* = 8.5 Hz, arom.), 8.32 (d, 2H, *J* = 8.5 Hz, arom.), 9.89 (s, 1H, OH). ^13^C-NMR (100 MHz, DMSO-*d*_6_): δ (ppm) 116.3, 119.5, 122.9, 124.4, 127.8, 130.2, 130.4, 131.37, 131.4, 138.2, 149.2, 152.9, 158.7 (C=O), 199.3 (C=S). LCMS (ESI): *m*/*z* 419.0/421.0 (97.1%, [M + H]^+^). Anal. Calc. for C_18_H_11_ClN_2_O_4_S_2_: C 51.61%; H 2.65%; N 6.69%. Found: C 51.80%; H 2.85%; N 6.80%.

2-{5-[(*Z*,2*Z*)-2-Chloro-3-(4-nitrophenyl)-2-propenylidene]-4-oxo-2-thioxothiazolidin-3-yl}-1-ethanesulfonic acid (**2g**). Yield: 83%, mp >260 °C. ^1^H-NMR (400 MHz, DMSO-*d*_6_): δ (ppm) 1.95 (t, 2H, *J* = 7.9 Hz, CH_2_), 3.47 (t, 2H, *J* = 7.9 Hz, CH_2_), 6.89 (s, 1H, CH=), 7.19 (s, 1H, CH=), 7.23 (d, 2H, *J* = 8.9 Hz, arom.), 7.51 (d, 2H, *J* = 8.8 Hz, arom.). ^13^C-NMR (100 MHz, DMSO-*d*_6_): δ (ppm) 41.7 (CH_2_), 47.6 (CH_2_), 124.3, 127.1, 130.3, 130.9, 131.3, 138.2, 140.3, 147.7, 167.1 (C=O), 194.3 (C=S). LCMS (ESI): *m*/*z* 432.8/435.0 (100%, [M + H]^+^). Anal. Calc. for C_14_H_11_ClN_2_O_6_S_3_: C 38.67%; H 2.55%; N 6.44%. Found: C 38.80%; H 2.45%; N 6.60%.

3-{5-[(*Z*,2*Z*)-2-Chloro-3-(4-nitrophenyl)-2-propenylidene]-4-oxo-2-thioxothiazolidin-3-yl}propanoic acid (**2h**). Yield: 75%, mp 254–256 °C. ^1^H-NMR (400 MHz, DMSO-*d*_6_): δ (ppm) 2.63 (t, 2H, *J* = 6.8 Hz, CH_2_), 4.22 (t, 2H, *J* = 6.8 Hz, CH_2_), 7.73 (s, 1H, CH=), 8.02 (s, 1H, CH=), 8.04 (d, 2H, *J* = 8.9 Hz, arom.), 8.32 (d, 2H, *J* = 8.9 Hz, arom.), 12.29 (br.s, 1H, COOH). LCMS (ESI): *m*/*z* 399.0/401.0/402.0 (100%, [M + H]^+^). Anal. Calc. for C_15_H_11_ClN_2_O_5_S_2_: C 45.17%; H 2.78%; N 7.02%. Found: C 45.00%; H 2.65%; N 6.90%.

6-{5-[(*Z*,2*Z*)-2-Chloro-3-(4-nitrophenyl)-2-propenylidene]-4-oxo-2-thioxothiazolidin-3-yl}hexanoic acid (**2i**). Yield: 75%, mp >220 °C. ^1^H-NMR (400 MHz, DMSO-*d*_6_): δ (ppm) 1.30 (quint, 2H, *J* = 6.7 Hz, CH_2_), 1.52 (quint, 2H, *J* = 7.1 Hz, CH_2_), 1.62 (quint, 2H, *J* = 6.7 Hz, CH_2_), 2.20 (quint, 2H, *J* = 7.0 Hz, CH_2_), 3.99 (quint, 2H, *J* = 6.9 Hz, CH_2_), 7.69 (s, 1H, CH=), 7.98 (s, 1H, CH=), 8.02 (d, 2H, *J* = 8.5 Hz, arom.), 8.30 (d, 2H, *J* = 8.5 Hz, arom.), 12.00 (s, 1H, COOH). ^13^C-NMR (100 MHz, DMSO-*d*_6_): δ (ppm) 24.5 (CH_2_), 26.1 (CH_2_), 26.5 (CH_2_), 33.8 (CH_2_), 44.5 (CH_2_), 124.3, 126.8, 130.2, 131.2, 131.3, 138.4, 140.2, 147.8, 167.4, 174.7 (C=O), 194.6 (C=S). LCMS (ESI): *m*/*z* 441.0/443.1 (100%, [M + H]^+^). Anal. Calc. for C_18_H_17_ClN_2_O_5_S_2_: C 49.03%; H 3.89%; N 6.35%. Found: C 49.10%; H 3.85%; N 6.40%.

2-{5-[(*Z*,2*Z*)-2-Chloro-3-(4-nitrophenyl)-2-propenylidene]-4-oxo-2-thioxothiazolidin-3-yl}-3-phenylpropanoic acid (**2j**). Yield: 70%, mp >220 °C. ^1^H-NMR (400 MHz, DMSO-*d*_6_): δ (ppm) 3.49 (t, 2H, CH_2_), 5.87 (br.s, 1H, CH), 7.10–7.25 (m, 5H, arom.), 7.71 (s, 1H, s, 1H, CH=), 8.00 (s, 1H, s, 1H, CH=), 8.02 (d, 2H, *J* = 8.0 Hz, arom.), 8.31 (d, 2H, *J* = 8.0 Hz, arom.), 13.59 (br.s, 1H, COOH). ^13^C-NMR (100 MHz, DMSO-*d*_6_): δ (ppm) 38.2 (CH_2_), 63.3 (CH), 129.1, 132.0, 133.5, 134.2, 134.7, 136.2, 137.1, 137.2, 141.6, 144.1, 152.6, 171.6 (C=O), 173.7 (C=O), 198.7 (C=S). LCMS (ESI): *m*/*z* 324.9/326.9 (100%, [M + H]^+^). Anal. Calc. for C_21_H_15_ClN_2_O_5_S_2_: C 53.11%; H 3.18%; N 5.90%. Found: C 53.00%; H 3.15%; N 5.80%.

2-{5-[(*Z*,2*Z*)-2-Chloro-3-(4-nitrophenyl)-2-propenylidene]-4-oxo-2-thioxothiazolidin-3-yl}succinic acid (**2k**). Yield: 68%, mp 220–222 °C. ^1^H-NMR (400 MHz, DMSO-*d*_6_): δ (ppm) 2.89 (d, 1H, *J* = 15.5 Hz, CH_2_), 3.23 (dd, *J* = 7.6, 15.6 Hz, 1H, CH_2_), 5.94 (br.s, 1H, CH), 7.75 (s, 1H, CH=), 8.02 (s, 1H, CH=), 8.04 (d, 2H, *J* = 8.9 Hz, arom.), 8.32 (d, 2H, *J* = 8.8 Hz, arom.), 12.68 (br.s, 2H, 2*COOH). ^13^C-NMR (100 MHz, DMSO-*d*_6_): δ (ppm) 33.0 (CH_2_), 53.2 (CH), 115.1 (C-Cl), 123.9, 129.6 (=CH), 131.0, 131.7 (=CH), 138.6, 139.7, 147.4, 166.5 (C=O), 168.6 (COOH), 171.2 (COOH), 193.5 (C=S). LCMS (ESI): *m*/*z* 442.8/444.7 (100%, [M + H]^+^). Anal. Calc. for C_16_H_11_ClN_2_O_7_S_2_: C 43.40%; H 2.50%; N 6.33%. Found: C 43.54%; H 2.48%; N 6.45%.

2-{5-[(*Z*,2*Z*)-2-Chloro-3-(4-nitrophenyl)-2-propenylidene]-4-oxo-2-thioxothiazolidin-3-yl}pentanedioic acid (**2l**). Yield: 72%, mp 205–207 °C. ^1^H-NMR (400 MHz, DMSO-*d*_6_): δ (ppm) 2.25–2.45 (m, 4H, CH_2_CH_2_), 5.59 (br.s, 1H, CH), 7.72 (s, 1H, CH=), 8.01 (s, 1H, CH=), 8.04 (d, 2H, *J* = 8.9 Hz, arom.), 8.32 (d, 2H, *J* = 8.9 Hz, arom.), 12.59 (br.s, 2H, 2*COOH). ^13^C-NMR (100 MHz, DMSO-*d*_6_): δ (ppm) 23.3 (CH_2_), 30.7 (CH_2_), 57.2 (CH), 119.3 (C-Cl), 124.3, 125.7 (=CH), 131.4, 131.8 (=CH), 138.8, 140.2, 147.8, 162.5 (C=O), 169.3 (COOH), 174.1 (COOH), 194.8 (C=S). LCMS (ESI): *m*/*z* 455.0/456.9 (100%, [M + H]^+^). Anal. Calc. for C_17_H_13_ClN_2_O_7_S_2_: C 44.69%; H 2.87%; N 6.13%. Found: C 44.56%; H 2.78%; N 6.05%.

### 3.3. Crystal Structure Determination of 6-{5-[(Z,2Z)-2-chloro-3-(4-nitrophenyl)-2-propenylidene]- 4-oxo-2-thioxothiazolidin-3-yl}hexanoic Acid Dimethylaminoformamide Solvate (***2i**·DMF*)

Compound **2i** was recrystallized from DMF by slow evaporation at room temperature.

Crystal data. C_18_H_17_ClN_2_O_5_S_2_, C_3_H_7_NO_2_, Mr = 514.00, monoclinic, space group *P*2_1_/*n*, *a* = 13.20068(11), *b* = 5.12876(4), *c* = 35.3537(3) Å, *β* = 94.7348(6)°, *V* = 2385.39(3) Å^3^, *Z* = 4 (Z’ = 1), *D*_calc_ = 1.431 g/cm^3^, *μ* = 3.425 mm^−1^, *T* = 130.0(1) K.

Data collection. An orange lath crystal (DMF) of 0.40 × 0.10 × 0.07 mm was used to record 18,412 (Cu Kα-radiation, *θ*_max_ = 76.22°) intensities on a Rigaku SuperNova Dual Atlas diffractometer [28] using mirror monochromatized Cu *K*α-radiation from a high-flux microfocus source (λ = 1.54178 Å). Accurate unit cell parameters were determined by least-squares techniques from the *θ* values of 12,519 reflections, *θ* range 3.47–76.02°. The data were corrected for Lorentz, polarization and for absorption effects [28]. The 4955 total unique reflections (*R*_int_ = 0.0175) were used for structure determination.

Structure solution and refinement. The structure was solved by a dual space algorithm (SHELXT) [29] and refined against F^2^ for all data (SHELXL) [30]. The position of the H atom bonded to the O atom was obtained from the difference Fourier map and was refined freely. The remaining H atoms were positioned geometrically and were refined within the riding model approximation: C–H = 0.98 Å (CH_3_), 0.99 Å (CH_2_), 0.95 Å (*Csp^2^*H), and *U*_iso_(H) = 1.2*U*_eq_(C) or 1.5*U*_eq_(C) for methyl H atoms. The methyl groups were refined as a rigid group, which were allowed to rotate. Final refinement converged with *R* = 0.0319 (for 4729 data with *F*^2^ > 4*σ*(*F*^2^), *w*R = 0.0864 (on *F*^2^ for all data), and *S* = 1.052 (on *F*^2^ for all data). The largest difference peak and hole was 0.281 and -0.275 eÅ^3^.

The molecular illustration was drawn using ORTEP-3 for Windows [31]. Software used to prepare material for publication was WINGX [31], OLEX2 [32], and PLATON [33].

The supplementary crystallographic data are deposited at the Cambridge Crystallographic Data Centre (CCDC), 12 Union ROAD, Cambridge CB2 1EZ (UK) [phone, (+44) 1223/336-408; fax, (+44) 1223/336-033; e-mail, deposit@ccdc.cam.ac.uk; World Wide Web, http://www.ccdc.cam.ac.uk, accessed on 18 April 2021 (deposition no. CCDC 2082064)].

### 3.4. In Vitro Evaluation of the Anticancer Activity According DTP NCI Protocol

Primary anticancer assay was performed on a panel of approximately sixty human tumor cell lines derived from nine neoplastic diseases, in accordance with the protocol of the Drug Evaluation Branch, National Cancer Institute, Bethesda [22,23,24,25]. Tested compounds were added to the culture at a single concentration (10^−5^ M) and the cultures were incubated for 48 h. End point determinations were made with a protein binding dye, sulforhodamine B (SRB). Results for each tested compound were reported as the percent of growth of the treated cells when compared to the untreated control cells. The percentage growth was evaluated spectrophotometrically versus controls not treated with test agents. The cytotoxic and/or growth inhibitory effects of the most active selected compounds were tested in vitro against the full panel of human tumor cell lines at concentrations ranging from 10^−4^ to 10^−8^ M. A 48 h continuous drug exposure protocol was followed, and an SRB protein assay was used to estimate cell viability or growth.

Using absorbance measurements (time zero (Tz), control growth in the absence of drug (C), and test growth in the presence of drug (Ti)), the percentage growth was calculated for each drug concentration. Percentage growth inhibition was calculated as:

[(Ti − Tz)/(C − Tz)] × 100 for concentrations for which Ti ≥ Tz(1)

[(Ti − Tz)/Tz] × 100 for concentrations for which Ti < Tz.(2)

Dose–response parameters (GI_50_, TGI, LC_50_) were calculated for each compound. Growth inhibition of 50% (GI_50_) was calculated from [(Ti − Tz)/(C − Tz)] × 100 = 50 (1), which is the drug concentration resulting in a 50% lower net protein increase in the treated cells (measured by SRB staining) as compared to the net protein increase seen in the control cells. The drug concentration resulting in total growth inhibition (TGI) was calculated from Ti = Tz. The LC_50_ (concentration of drug resulting in a 50% reduction in the measured protein at the end of the drug treatment as compared to that at the beginning) indicating a net loss of cells following treatment was calculated from [(Ti − Tz)/Tz] × 100 = −50 (2). Values were calculated for each of these parameters if the level of activity was reached; however, if the effect was not reached or was excessive, the value for that parameter was expressed as more or less than the maximum or minimum concentration tested. The lowest values were obtained with the most sensitive cell lines. Compounds having GI_50_ values ≤ 100 μM were declared to be active.

### 3.5. Cell Viability Assay (AGS, DLD-1, MCF-7 and MDA-MB-231 Cell Lines; Human Blood Lymphocytes)

The assay was performed by using 3-(4,5-dimethylthiazole-2-yl)- 2,5-diphenyltetrazolium bromide (MTT). Confluent cells, cultured for 24 h with 0.1, 1, 5, 10, 20, 30, and 100 µM concentrations of studied compounds in 24-well plates were washed with PBS. MTT was dissolved in PBS, and 25 µL were added to each well. Plates were incubated for 4 h at 37 °C in 5% CO_2_ in an incubator. The medium with MTT was removed, and 1 mL of DMSO was added to the attached cells. Furthermore, cells were incubated for 5–10 min in RT and then 10 µl of Sorensen buffer was added to each well. The absorbance of converted dye in living cells was measured at a wavelength of 570 nm. The cell viability of breast cancer cells, gastric cancer cells, and human colon cancer cells cultured in the presence of ligands was calculated as percent of control cells.

### 3.6. Isolation of Human Blood Lymphocytes and Their Activation

First, 20 mL of venous blood was taken from volunteers (Ethical protocol number 2, 27 January 2019) and collected in the presence of 200 μL of undiluted fresh heparin (1/100). Sterile blood was diluted 2 times with 0.9% NaCl under the sterile conditions. Isolation of lymphocytes was performed in a density gradient of ficol-verografin using the protocol of the manufacturer (Lympoprep, NYCOMED PHARMA AS, Oslo Norway). The resulting lymphocytes were resuspended in the RPMI-1640 medium and cultured for several days (up to 10 days). To separate the lymphocytes from the monocytes, cell suspension was left for 24 h. After 24 h of culture, monocytes were attached, while lymphocytes were transferred to a fresh Falcon tube (15 mL). To stimulate the proliferation of lymphocytes, they were cultured on CD3+ antibody-coated plastic plate in the RPMI-1640 medium supplemented with 20% FBS.

## 4. Conclusions

In the presented paper, new 5-[(*Z*,2*Z*)-2-chloro-3-(4-nitrophenyl)-2-propenylidene]- 4-thiazolidinones (*Ciminalum*-thiazolidinone hybrid molecules) are described. NCI 60-Cell-line antitumor activity assay allowed identifying a highly active compound **2h** with the mean GI_50_ 1.57 μM and TGI 13.3 μM with a certain sensitivity profile in the GI_50_ concentration range of < 0.01–0.02 μM toward leukemia (MOLT-4, SR), colon cancer (SW-620), CNS cancer (SF-539) and melanoma (SK-MEL-5) cell lines. High cytotoxicity of 5-[(Z,2*Z*)-2-chloro-3-(4-nitrophenyl)-2-propenylidene]-2-thioxo-4-thiazolidinone-3-carboxylic acids against cell lines of gastric cancer (AGS), human colon cancer (DLD-1), and breast cancers (MCF-7 and MDA-MB-231) was established. The hit compounds **2f**, **2i**, **2g**, and **2h** have been found to have low toxicity toward normal human blood lymphocytes and a fairly wide therapeutic range—TI for leukemia panel > 353.36 (**2f**), 376.14 (**2h**) and 16.60 (**2j**). The SAR analysis allowed confirming the crucial role of 2-chloro-3-(4-nitrophenyl)prop-2-enylidene (*Ciminalum*) substituent in position 5 for 4-thiazolidinones and establish the dependence of the anticancer activity of the synthesized compounds on the nature of the substituents in *N3* position of the core heterocycle. Further investigations on the *Ciminalum*–thiazolidinone hybrid molecules could lead to more potent compounds as promising candidates for the development of new anticancer chemotherapy. The levels of their anticancer activity cause the need for the in-depth study of their mechanisms of action.

## Figures and Tables

**Table 1:** Hydrogen-bond geometry (Å, °) for **2i**·DMF.

*D*—H∙∙∙*A*	*D*—H	H∙∙∙*A*	*D*∙∙∙*A*	*D*—H∙∙∙*A*
O14—H14∙∙∙O29	0.95 (3)	1.63 (3)	2.5758 (16)	171 (3)
C11—H11A∙∙∙O13 ^i^	0.99	2.55	3.5287 (19)	172
C11—H11A∙∙∙O15 ^ii^	0.99	2.47	3.135 (2)	125
C21—H22∙∙∙C118	0.95	2.51	3.2049 (15)	130
C22—H22∙∙∙S6 ^iii^	0.95	2.75	3.6734 (16)	164
C25—H25∙∙∙O29 ^iv^	0.95	2.50	3.4332 (18)	169

Symmetry codes: (i) *x*, 1 + *y*, *z*; (ii) 1/2 − *x*, 1/2 + *y*, 1/2 − *z*; (iii) 1 − *x*, 2 − *y*, 1 − *z*; (iv) 1/2 − *x*, −1/2 + *y*, 1/2 − *z*.

**Table 2:** Influence of compounds **2b**, **2c**, **2f**, **2h**, and **2j** on the growth of individual tumor cell lines.

Cell line/comp.	2b	2c	2f	2h	2j
	GI_50_/TGI/LC_50_, μM	GI_50_/TGI/LC_50_, μM	GI_50_/TGI/LC_50_, μM	GI_50_/TGI/LC_50_, μM	GI_50_/TGI/LC_50_, μM
**Leukemia**					
CCRF-CEM	1.97/21.5/>50	4.42/35.3/>100			2.92/>100/>100
HL-60(TB)	1.21/14.4/>50	5.32/23.3/87.2	0.485/4.48/>100	0.347/1.87/>100	
K-562	1.44/42.5/>50	3.98/25.5/>100	0.521/>100/>100	0.212/2.83/>100	3.01/>100/>100
MOLT-4	1.74/14.2/>50	3.20/8.95/52.5	0.389/>100/>100	0.016/19.7/>100	3.14/>100/>100
RPMI-8226	1.54/20.6/>50	2.38/6.32/>100	<0.01/0.264/>100	0.138/1.30/>100	3.22/>100/>100
SR	1.18/7.96/>50	3.67/12.0/>100	0.01/0.138/>100	<0.01/1.41/>100	2.45/>100/>100
**Non-Small Cell Lung Cancer**					
A549/ATCC	8.18/>50/>50	18.9/42.6/96.1	2.25/6.47/>100	1.76/7.62/>100	3.46/>100/>100
EKVX	2.76/>50/>50	23.7/47.7/96.1	2.37/16.2/>100	0.334/4.01/55.6	2.59/7.29/>100
HOP-62	15.4/33.1/>50	17.2/39.7/91.9	2.61/5.22/11.6	6.54/19.9/52.8	2.31/6.75/>100
HOP-92	8.48/>50/>50		8.53/39.4/>100	4.81/18.3/50.7	2.36/5.71/>100
NCI-H226	6.28/20.0/> 50	12.0/31.8/84.2			1.58/3.27/6.76
NCI-H23	3.27/13.4/45.5	16.6/31.6/60.3	1.48/3.11/6.52	1.44/3.22/7.19	2.13/4.86/>100
NCI-322M	6.51/18.8/>50	87.6/>100/>100	2.49/9.02/>100	2.83/10.9/63.4	>100/>100/>100
NCI-H460	3.95/18.6/>50	15.0/32.3/69.5	0.754/>100/>100	0.804/3.32/>100	2.19/>100/>100
NCI-H522	3.19/13.1/42.3	16.3/32.9/66.2			1.72/4.47/29.5
**Colon Cancer**					
Colo 205	4.04/17.4/>50	56.2/>100/>100	4.34/>100/>100	0.350/>100/>100	2.45/>100/>100
HCC-2998	5.43/11.7/25.0				1.96/3.50/6.27
HCT-116	2.93/11.0/33.7	2.73/6.51/23.3	1.27/2.96/6.92	0.270/1.19/>100	2.01/5.47/56.7
HCT-15	1.79/8.81/25.4	16.6/33.0/65.7	0.409/>100/>100	0.230/2.96/>100	1.92/4.58/>100
HT-29	1.48/9.48/>50		2.14/>100/>100	1.25/2.77/>100	2.84/8.47/>100
KM12	5.27/14.6/40.4	20.6/59.1/>100	2.85/>100/>100	0.639/29.5/>100	2.06/4.35/9.16
SW-620	1.94/7.62/23.5	7.24/>100/>100	0.037/1.52/>100	<0.01/3.25/>100	2.10/4.39/>100
**CNS Cancer**					
SF-268	5.24/18.9/>50	17.0/41.3/>100	2.55/34.5/>100	2.12/9.74/>100	2.21/>100/>100
SF-295	8.46/37.9/>50	18.0/37.3/77.1	2.26/13.8/>100	3.36/21.6/>100	2.59/7.85/>100
SF-539	9.39/23.1/>50	25.9/57.4/>100	0.0252/0.242/28.1	<0.01/0.267/25.8	2.74/>100/>100
SNB-19	8.10/21.4/>50	48.3/>100/>100	10.0/32.0/97.5	0.658/26.3/83.7	1.85/> 100/>100
SNB-75	7.59/23.5/>50		25.1/>100/>100	12.5/46.0/>100	1.96/5.37/>100
U251	3.09/9.73/22.1	8.03/24.5/65.8	0.368/3.06/38.9	0.149/2.00/>100	1.72/3.52/7.21
**Melanoma**					
LOX IMVI	6.60/15.4/36.0	13.0/28.8/63.8	1.17/3.70/>100	0.161/0.515/>100	1.64/3.26/6.46
MALME-3M	4.93/14.9/44.6	25.0/60.4/>100	6.53/27.6/94.5	9.70/61.9/>100	2.13/4.82/>100
M14	8.87/32.2/>50	16.2/35.7/78.5	1.90/3.73/7.32	1.66/3.58/7.74	2.53/>100/>100
SK-MEL-2	8.07/26.9/>50	19.0/36.4/69.8		0.802/4.93/65.1	1.87/4.50/>100
SK-MEL-28	5.87/12.3/25.9		4.49/47.7/>100	3.59/23.6/89.5	2.16/4.68/11.6
SK-MEL-5	3.82/10.6/24.0	19.4/38.3/75.6	<0.01/<0.01/3.41	0.0193/0.0849/1.88	1.68/3.06/5.60
UACC-62	5.75/12.5/27.2	13.6/28.1/58.0	1.69/3.85/8.78	1.23/2.97/7.18	2.00/4.85/30.2
**Ovarian Cancer**					
IGROV1	7.28/21.1/>50	13.3/28.0/58.9	1.63/6.13/>100	0.794/3.68/43.1	2.99/>100/>100
OVCAR-3	1.65/8.08/26.6	0.821/12.7/41.1	0.977/41.8/>100	0.135/6.05/66.6	1.76/3.51/6.98
OVCAR-4	1.95/11.7/>50		2.63/>100/>100	2.66/14.9/56.2	3.21/>100/>100
OVCAR-5	11.1/19.5/32.4	15.1/36.2/86.6			2.82/7.91/>100
OVCAR-8	5.08/15.6/47.9	1.98/4.08/8.38	1.14/22.9/>100	0.244/11.5/>100	3.13/>100/>100
SK-OV-3	13.0/>50/>50	59.6/>100/>100	10.2/41.5/>100	6.83/27.4/77.6	4.13/>100/>100
**Renal Cancer**					
786-0	9.40/18.8/37.6	12.1/30.7/77.8	2.04/3.83/7.18	1.13/2.47/5.42	2.10/5.01/>100
A498	4.57/12.2/30.9	13.0/29.6/67.3			1.65/3.24/6.37
ACHN	3.64/11.8/31.7	15.6/29.7/56.4	1.70/3.51/7.27	1.02/2.42/5.76	1.80/3.30/6.04
CAKI-1	7.14/25.2/>50	3.11/11.2/>100	4.44/>100/>100	0.485/6.14/47.1	2.28/5.09/16.7
RXF 393				0.105/0.341/1.73	1.54/3.02/5.93
SN12C	4.11/18.0/>50	29.0/>100/>100	1.98/4.41/>100	1.05/2.99/8.48	2.84/>100/>100
TK-10	10.6/23.2/>50	28.0/69.6/>100	4.87/29.0/>100	2.33/16.9/92.0	2.43/>100/>100
UO-31	4.12/21.8/>50	17.5/32.2/59.1	2.08/3.98/>100	0.812/2.43/6.31	1.59/3.10/6.05
**Prostate Cancer**					
PC-3	4.68/>50/>50	8.10/45.8/>100	2.29/84.8/>100	0.712/13.7/41.9	2.53/7.98/>100
DU-145	7.09/14.2/28.4	21.7/52.8/>100	0.666/5.81/50.1	0.421/3.48/52.3	2.06/4.56/11.7
**Breast Cancer**					
MCF-7	2.14/38.0/>50	4.08/19.8/83.7	0.401/28.0/>100	0.239/14.4/>100	2.38/10.7/>100
NCI/ADR-Res	5.71/18.3/>50	6.55/20.8/61.7	2.17/6.13/>100	0.407/2.11/6.43	
MDA-MB-231/ATCC	8.16/28.1/>50	10.0/35.4/>100	2.18/12.2/92.1	1.08/4.86/25.9	3.35/>100/>100
HS 578T	1.42/12.1/>50	29.5/>100/>100			4.37/>100/>100
MDA-MB-435	8.57/47.9/>50	20.3/41.9/86.3	3.50/7.71/>100	1.25/4.12/29.3	
BT-549	1.47/4.74/27.2	27.3/76.0/>100	1.55/7.71/>100	0.247/0.920/36.3	1.79/4.17/>100
T-47D	3.21/>50/>50	24.5/65.3/>100	1.55/7.71/>100	0.363/>100/>100	1.50/3.71/>100
MDA-MB-468					1.39/3.28/7.71

**Table 3:** Influence of **2b**, **2c**, **2f**, **2h**, and **2j** on the growth of tumor panels (GI_50_, TGI, LC_50_) and selectivity index (SI) values.

Compound/Disease		2b		2c		2f		2h		2j	
		MG_MID, μM	SI	MG_MID, μM	SI	MG_MID, μM	SI	MG_MID, μM	SI	MG_MID, μM	SI
Leukemia	GI_50_	1.51	3.17	3.83	4.70	0.283	**9.89**	0.145	**10.83**	2.95	1.39
	TGI	20.2	1.00	18.6	2.32	41.0	0.78	5.42	2.45	>100	<0.41
	LC_50_	>50	<0.87	90	0.91	>100	<0.81	>100	<0.65	>100	<0.74
Non-Small Cell Lung Cancer	GI_50_	3.79	1.26	25.9	0.69	2.93	0.96	2.65	0.59	13.1	0.31
	TGI	29.7	0.68	44.8	0.96	26.6	1.21	9.61	1.38	36.9	1.10
	LC_50_	48.6	0.90	83.0	0.98	74.0	1.09	61.4	1.06	92.9	0.80
Colon Cancer	GI_50_	3.27	1.46	20.7	0.87	1.84	1.52	0.458	3.43	2.19	1.87
	TGI	11.5	1.77	59.7	0.72	67.4	0.48	23.3	0.57	18.7	2.17
	LC_50_	35.4	1.23	77.8	1.05	84.5	0.96	>100	<0.65	67.4	1.10
CNS Cancer	GI_50_	6.98	0.69	23.4	0.77	6.72	0.42	3.13	0.50	2.18	1.88
	TGI	22.4	0.91	52.1	0.83	30.6	1.05	17.7	0.75	3.52	**11.53**
	LC_50_	45.4	0.96	88.6	0.92	77.4	1.04	84.9	0.77	7.21	**10.25**
Melanoma	GI_50_	5.57	0.86	17.7	1.02	2.63	1.06	2.45	0.64	2.00	2.05
	TGI	16.1	1.26	38.0	1.13	14.4	2.23	13.9	0.96	17.9	2.27
	LC_50_	36.8	1.18	74.3	1.10	52.3	1.54	53.1	1.22	50.6	1.46
Ovarian Cancer	GI_50_	6.68	0.72	18.2	0.99	3.32	0.84	2.13	0.74	3.00	1.36
	TGI	21.0	0.97	36.2	1.19	42.5	0.76	12.7	1.05	68.6	0.59
	LC_50_	42.8	1.02	59.0	1.38	>100	<0.81	68.7	0.95	84.5	0.87
Renal Cancer	GI_50_	6.23	0.77	16.9	1.07	2.85	0.98	0.99	1,59	2.03	2.01
	TGI	18.7	1.09	43.3	1.00	24.1	1.34	4.81	2.77	27.8	1.46
	LC_50_	42.9	1.01	80.1	1.02	69.1	1.17	23.8	2.73	42.6	1.73
Prostate Cancer	GI_50_	5.89	0.81	14.9	1.21	1.48	1.89	0.567	2.77	2.30	1.78
	TGI	32.1	0.63	45.3	0.95	45.3	0.71	8.59	1.55	6.27	6.48
	LC_50_	39.2	1.11	75.1	1.09	75.1	1.08	47.1	1.38	55.9	1.32
Breast Cancer	GI_50_	4.38	1.09	17.5	1.03	1.89	1.48	0.598	2.63	24.6	0.17
	TGI	28.4	0.71	51.3	0.84	11.6	2.78	21.1	0.63	37.0	1.10
	LC_50_	46.7	0.93	90.2	0.91	98.7	0.82	49.6	1.31	84.6	0.87
60 lines MG_MID	GI_50_	**4.79**		**18.0**		**2.80**		**1.57**		**4.09**	
	TGI	**20.3**		**43.1**		**32.2**		**13.3**		**40.6**	
	LC_50_	**43.5**		**81.7**		**80.8**		**65.0**		**73.9**	

**Table 4:** Influence of compounds **2a–e, 2g**, **2h**, **2k**, and **2l** on the growth of AGS, DLD-1, MCF-7, and MDA-MB-231 cell lines.

Compound	Cell line, GI_50_, μM			
	AGS	DLD-1	MCF-7	MDA-MB-231
**2a**	18.71	13.98	18.03	13.89
**2b**	7.86	8.39	4.79	10.56
**2c**	4.43	6.34	3.60	1.59
**2d**	4.08	5.47	4.45	3.11
**2e**	17.05	16.00	17.92	15.83
**2g**	17.99	27.49	26.40	16.84
**2h**	2.69	3.67	3.62	1.63
**2i**	3.20	9.22	1.73	0.95
**2k**	13.05	10.00	18.08	15.30
**2l**	12.57	9.19	21.23	17.50

**Table 5:** Therapeutic index (TI) for compounds **2f**, **2h**, and **2j** regarding diversity leukemia cell lines.

Compound	Leukemia Cell Line TI (Therapeutic Index)					
	HL-60(TB)	K-562	MOLT-4	RPMI-8226	SR	Leukemia Panel
**2f**	>206.19	>191.94	>257.07	>10,000	>10,000	**>353.36**
**2h**	157.18	257.26	3408.75	395.22	5454	**376.14**
**2j**	n/a	16.27	15.59	15.21	19.99	**16.60**

## Data Availability

Data available in a publicly accessible repository.

## Supplementary Materials

The following are available online, Figures S1–S32: Copies of NMR and LCMS spectra of compounds **2a–l**.

## Sample Availability

Samples of the compounds **2a–l** are available from the authors.
