# Supplementary material for: Synthesis and Anticancer Activity Evaluation of 5-[2-Chloro-3-(4-nitrophenyl)-2-propenylidene]-4-thiazolidinones
Source: Molecules. 2021 May 20;26(10):3057. doi: 10.3390/molecules26103057 (PMC8161026; doi:10.3390/molecules26103057)
Supplement: Supplementary file 1 [file molecules-26-03057-s001.zip › molecules-1207887-supplementary-done.pdf]

molecules

Supplementary information

Synthesis and Anticancer Activity Evaluation of 5-[-2-Chloro-3-(4-nitrophenyl)-2-propenylidene]-4-thiazolidinones

Kamila Buzun [1], Anna Kryshchyshyn-Dylevych [2], Julia Senkiv [3], Olexandra Roman [2], Krzysztof Bielawski [4], Anna Bielawska [1] and Roman Lesyk [2,5*]

[1] Department of Biotechnology, Medical University of Bialystok, Jana Kilińskiego 1, Bialystok, 15-089, Poland; kamila.buzun@umb.edu.pl (K.B.), anna.bielawska@umb.edu.pl (A.B.)
[2] Department of Pharmaceutical, Organic and Bioorganic Chemistry, Danylo Halytsky Lviv National Medical University, Pekarska 69, Lviv, 79010, Ukraine; kryshchyshyn.a@gmail.com (A.K.-D.), lesia_roman@ukr.net (O.R.), dr_r_lesyk@org.lviv.net (R.L.)
[3] Institute of Cell Biology of National Academy of Sciences of Ukraine, 14/16 Drahomanov Str., Lviv 79005, Ukraine; yu.senkiv@gmail.com
[4] Department of Synthesis and Technology of Drugs, Medical University of Bialystok, 15-089 Bialystok, Poland; kbiel@umb.edu.pl
[5] Department of Public Health, Dietetics and Lifestyle Disorders, Faculty of Medicine, University of Information Technology and Management in Rzeszow, 35-225 Rzeszow, Poland
* Correspondence: dr_r_lesyk@org.lviv.net; Tel.: +380322755966

Supplementary data: The NMR spectra of compounds 2a-l

Citation: Buzun, K.; Kryshchyshyn-Dylevych, A.; Senkiv, J.; Roman, O.; Bielawski, K.; Bielawska, A.; Lesyk, R. Synthesis and Anticancer Activity Evaluation of 5-[-2-Chloro-3- (4-nitrophenyl)-2-propenylidene]-4-thiazolidinones. *Molecules* **2021**, *26*, x. https://doi.org/10.3390/xxxxx

Received: date
Accepted: date
Published: date

Publisher's Note: MDPI stays neutral with regard to jurisdictional claims in published maps and institutional affiliations.

Copyright: © 2021 by the authors. Submitted for possible open access publication under the terms and conditions of the Creative Commons Attribution (CC BY) license (http://creativecommons.org/licenses/by/4.0/).

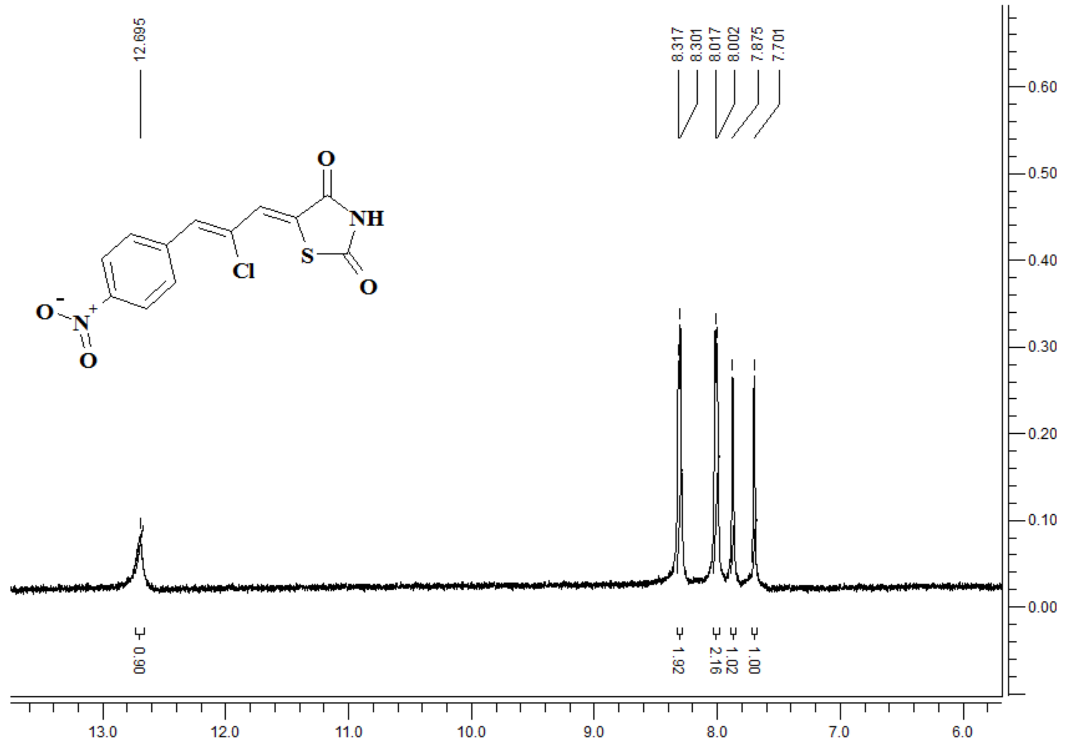

Figure S1. ^{1}H NMR spectrum **2a.**

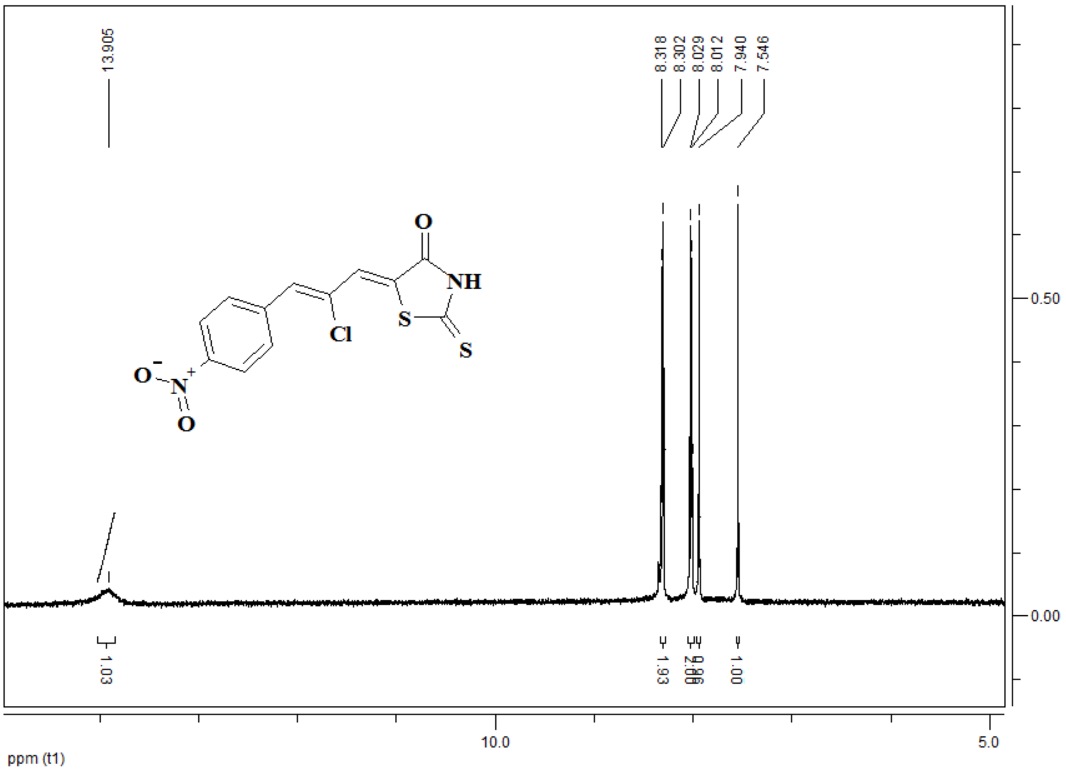

Figure S2. ^{1}H NMR spectrum **2b.**

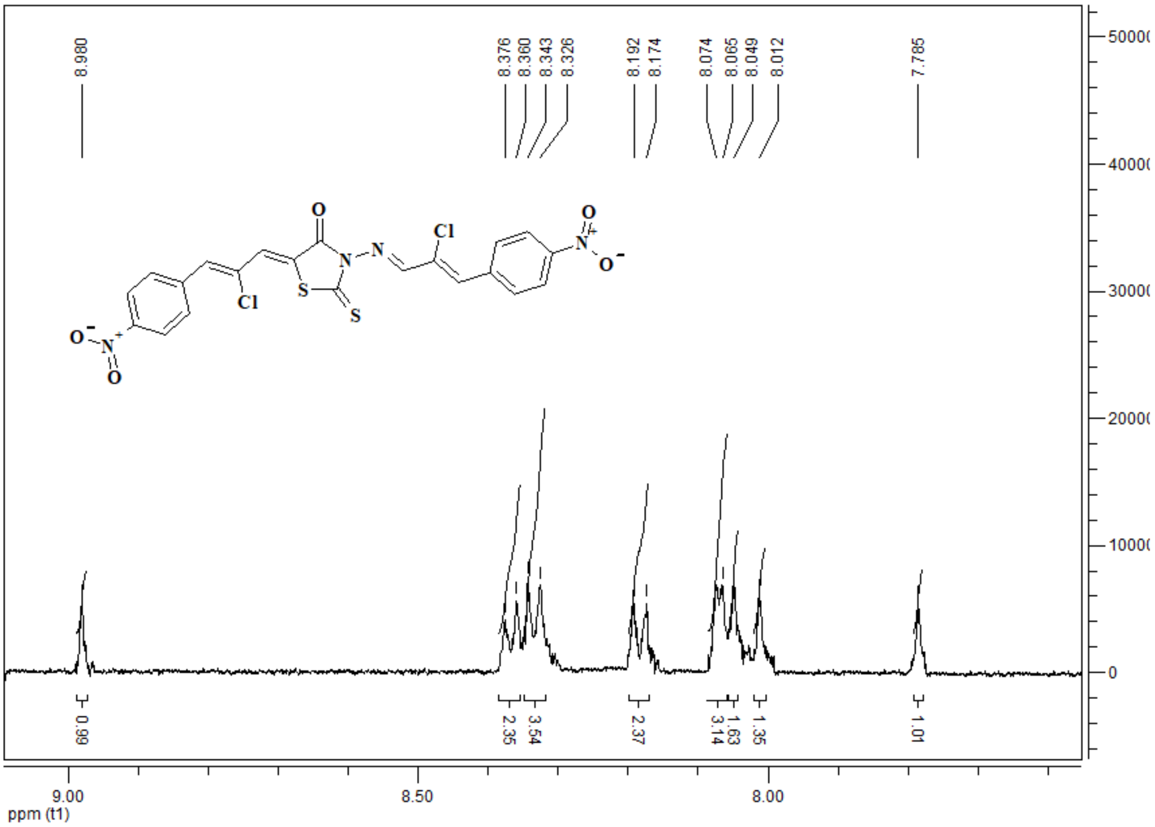

Figure S3. ^{1}H NMR spectrum **2c.**

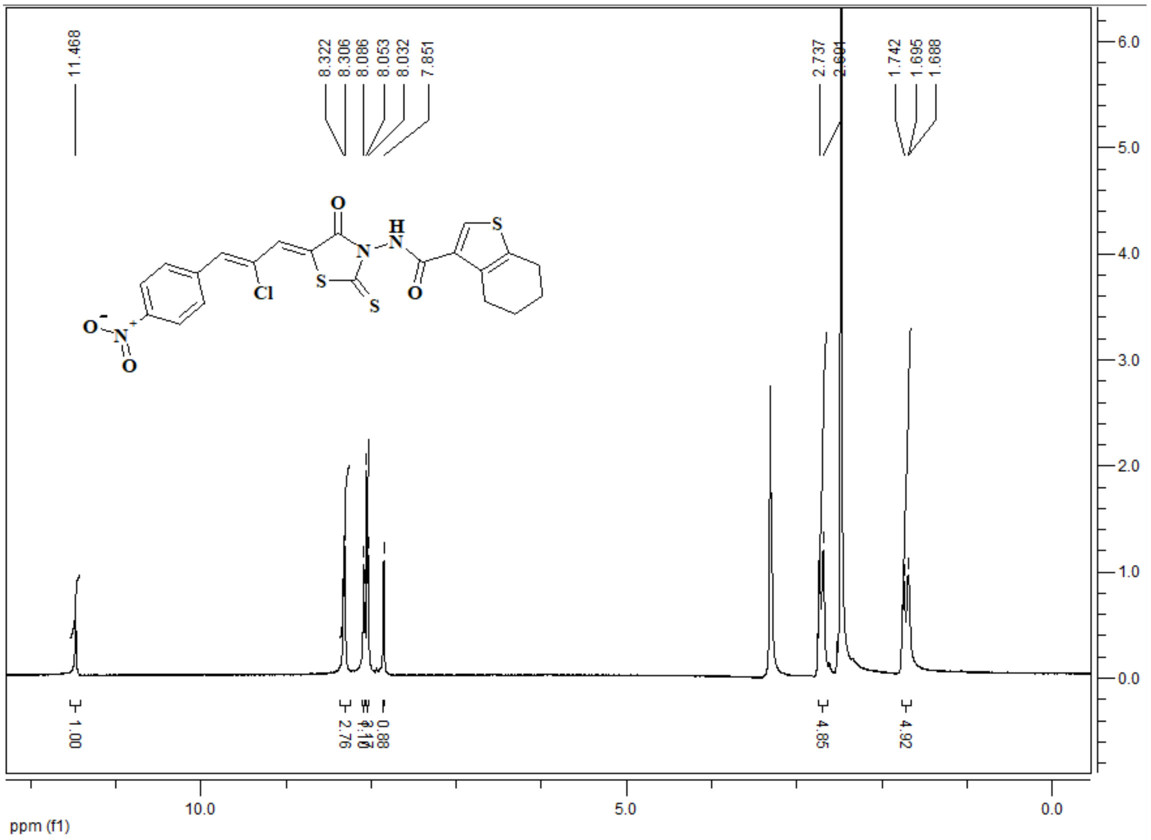

Figure S4. ^{1}H NMR spectrum **2d.**

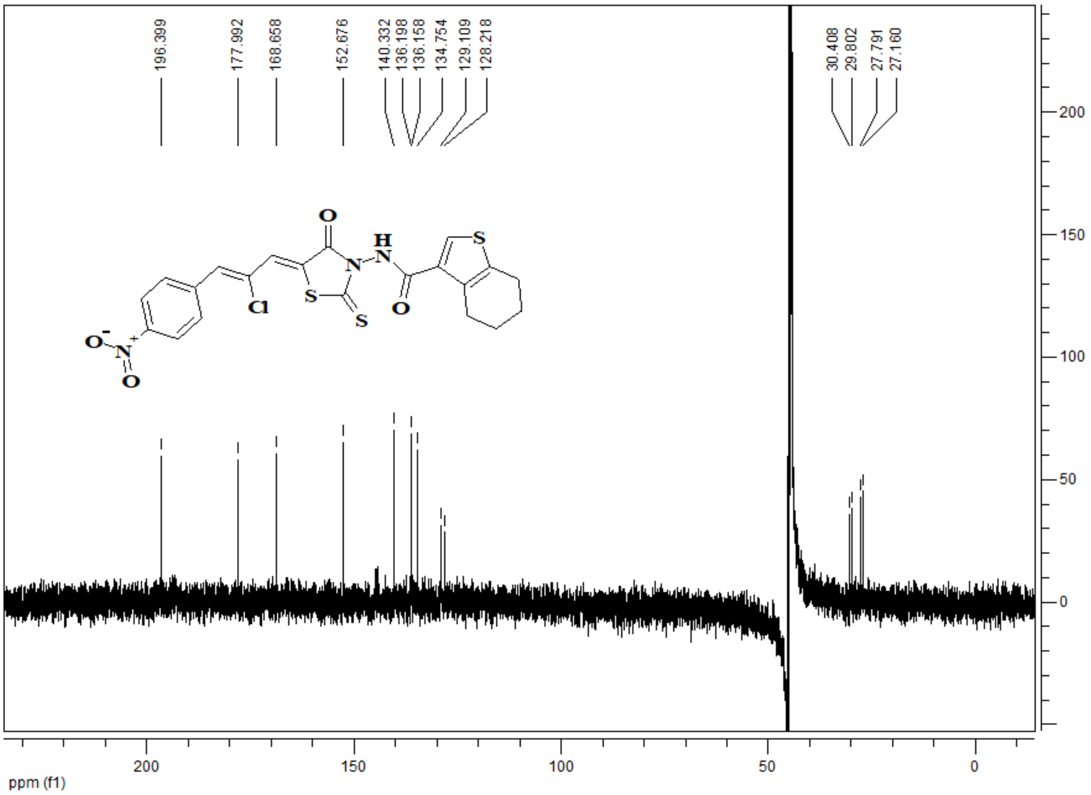

Figure S5. ^{13}C NMR spectrum **2d.**

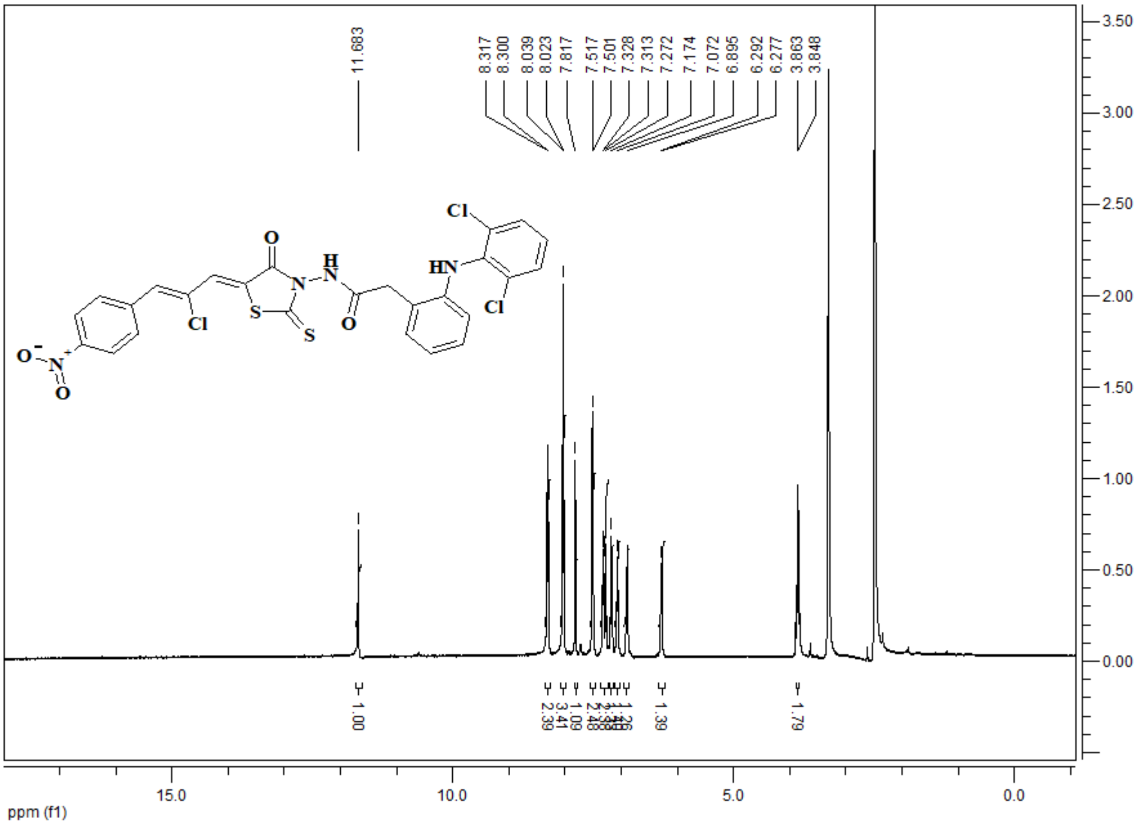

Figure 6. ^{1}H NMR spectrum **2e.**

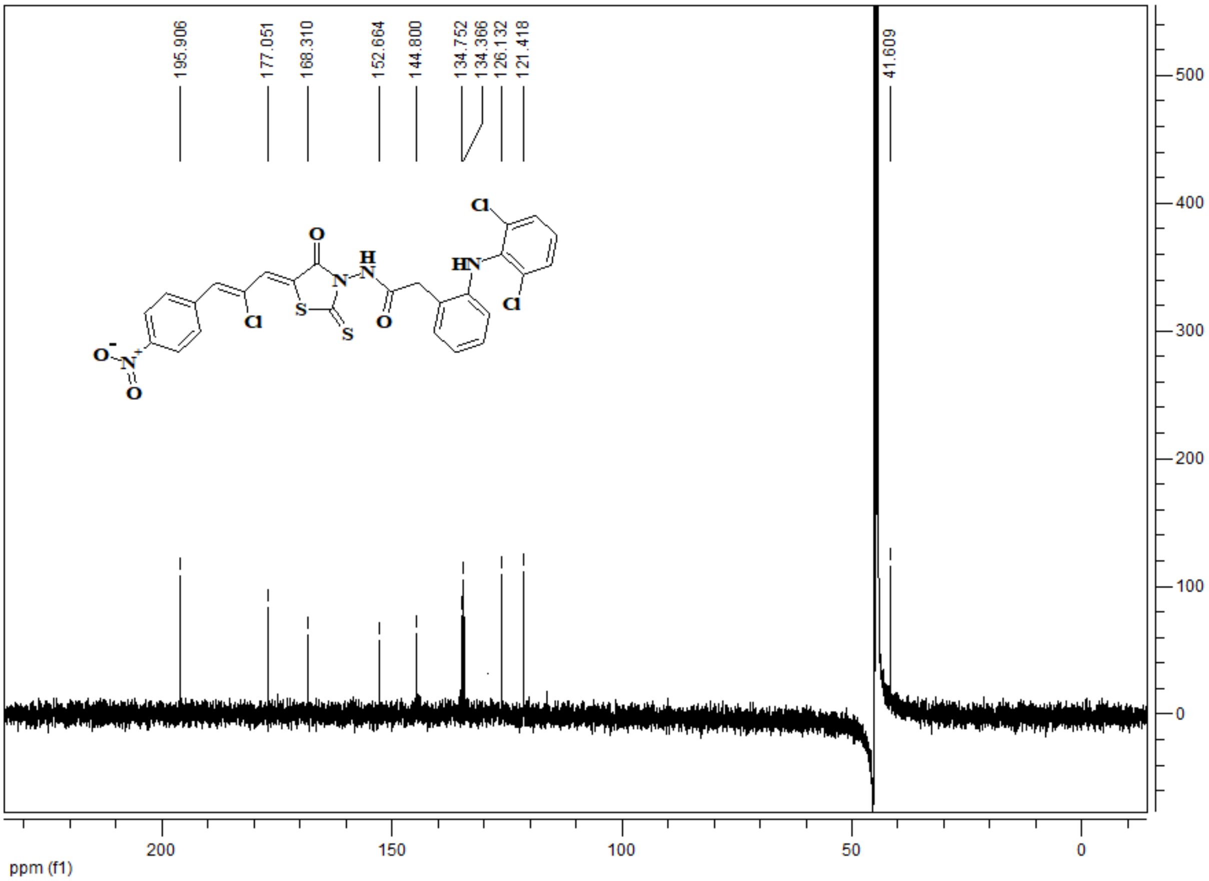

Figure S7. ^{13}C NMR spectrum **2e.**

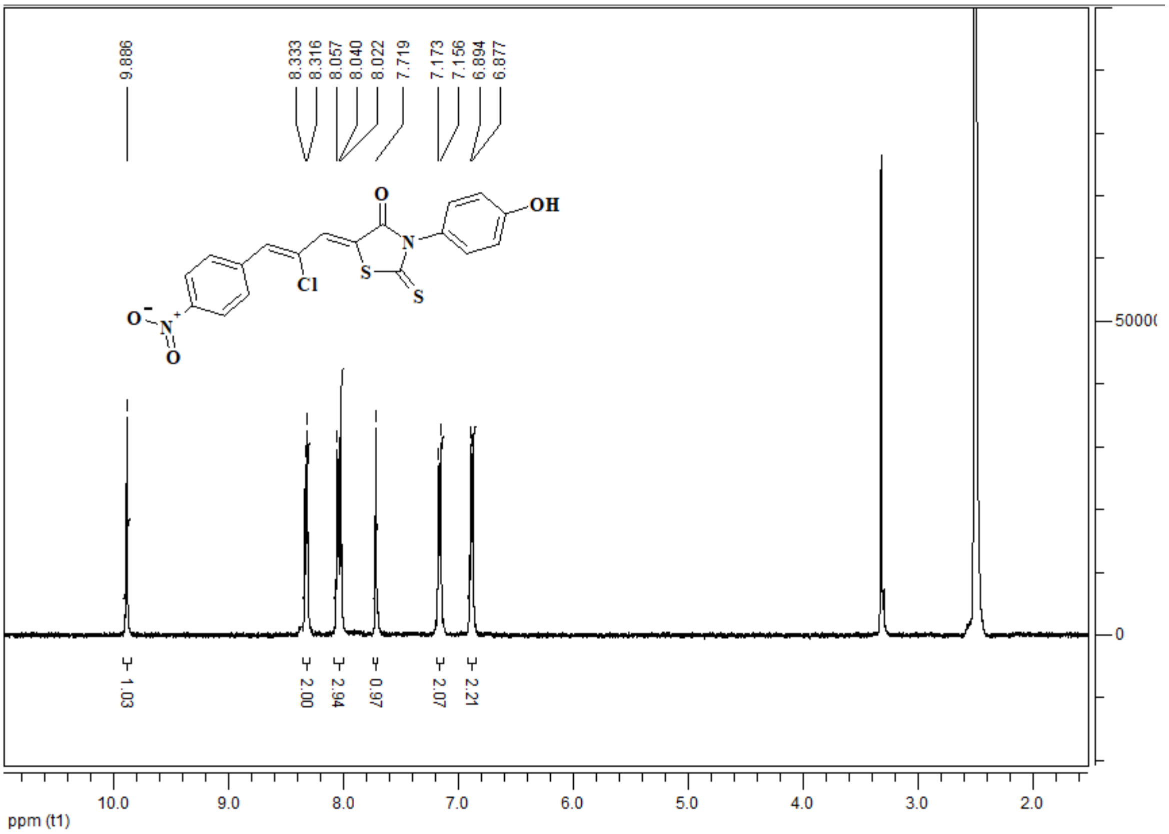

Figure S8. ^{1}H NMR spectrum **2f.**

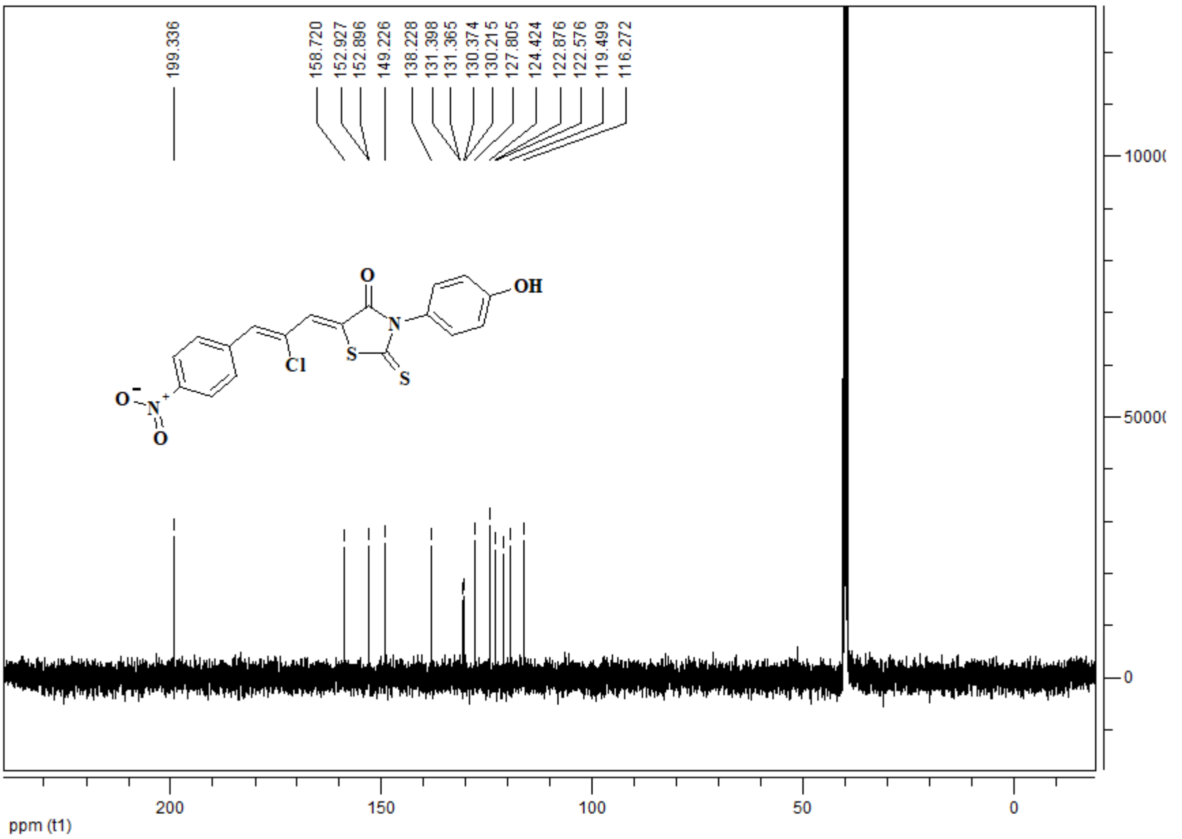

Figure S9. ^{13}C NMR spectrum **2f.**

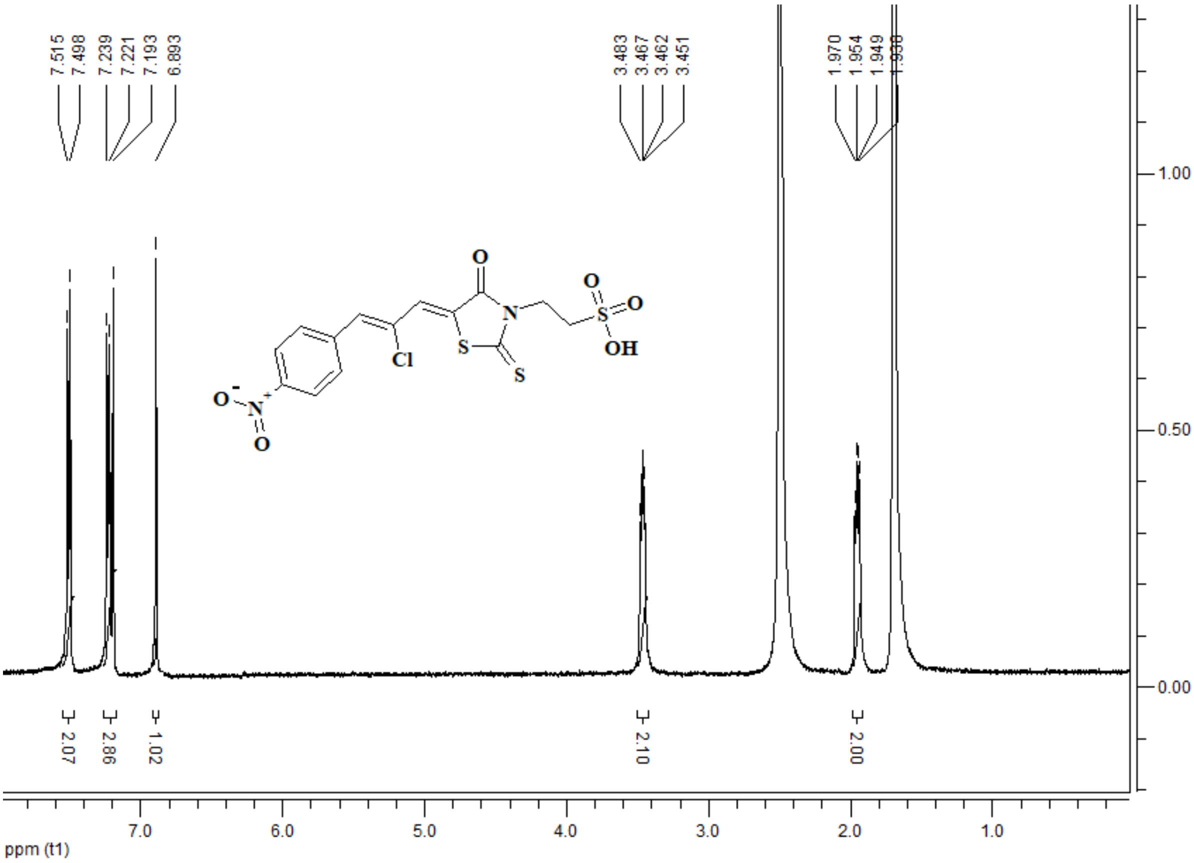

Figure S10. ^{1}H NMR spectrum **2g.**

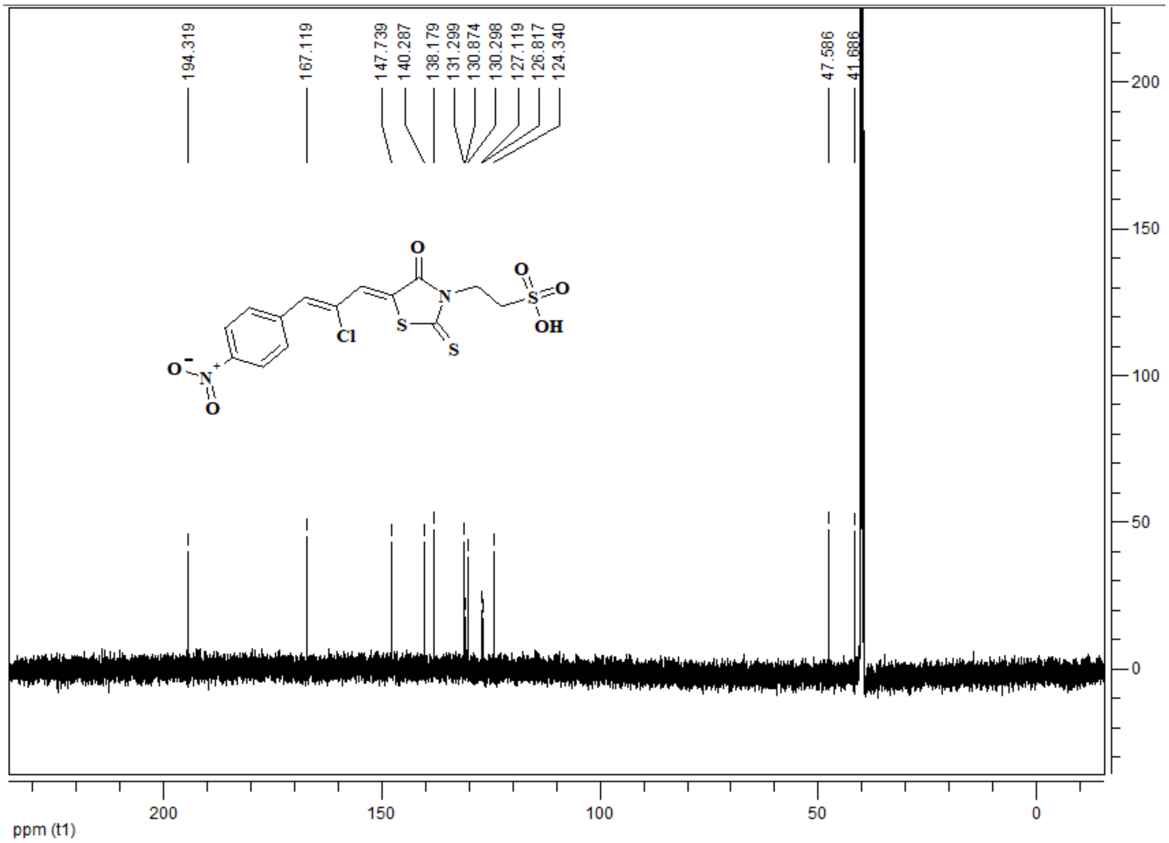

Figure S11. ^{13}C NMR spectrum **2g.**

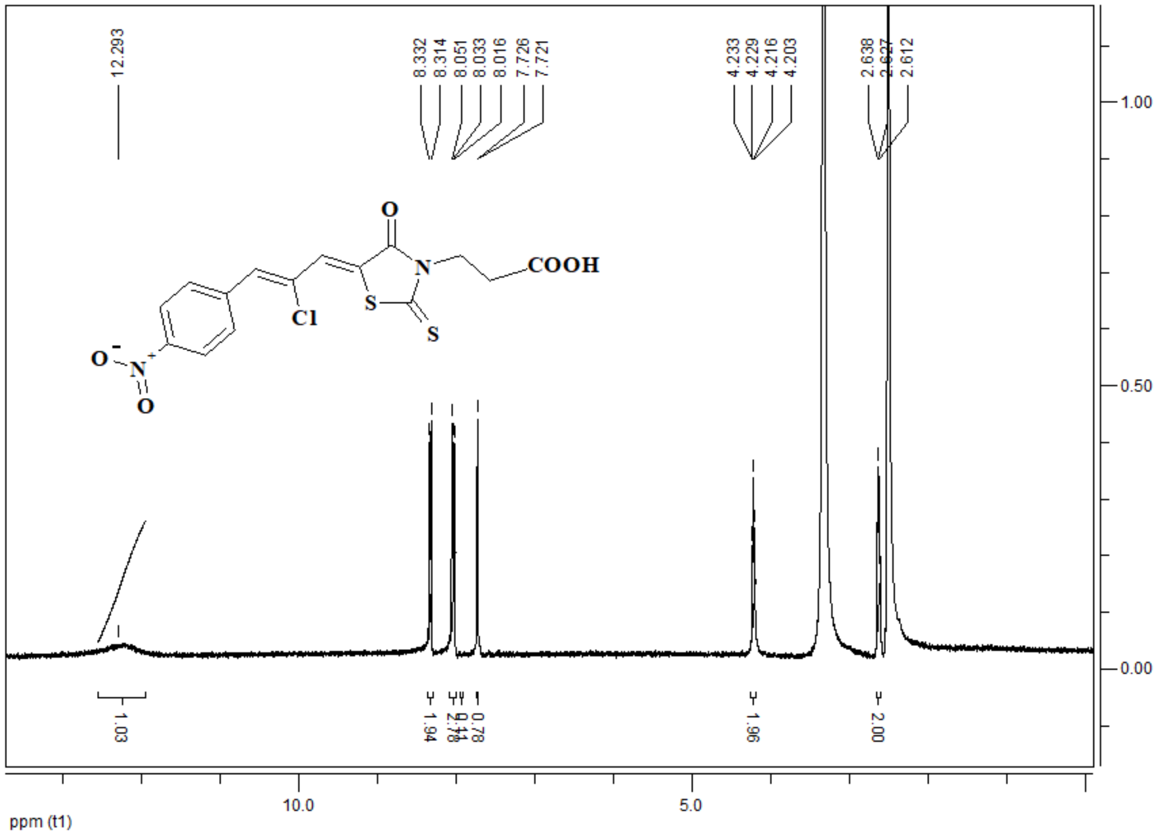

Figure S12. ^{1}H NMR spectrum **2h.**

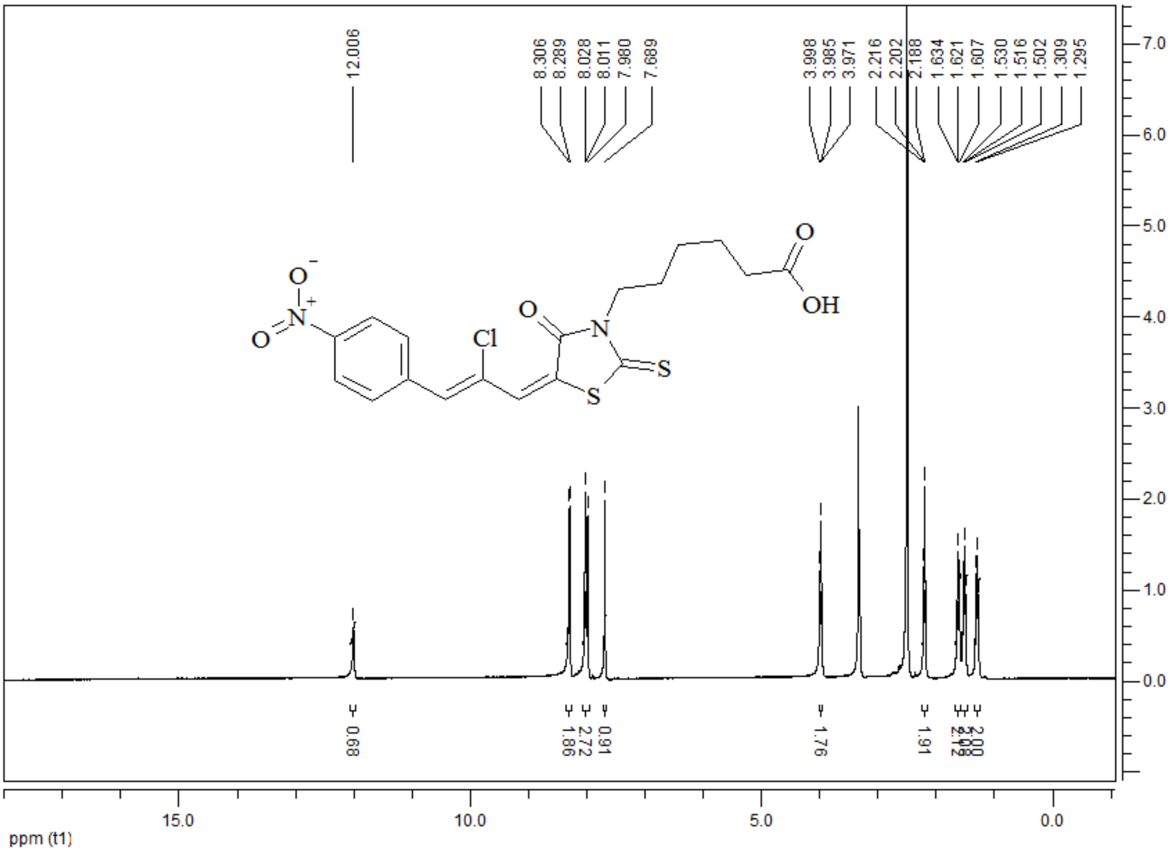

Figure S13. ^{1}H NMR spectrum **2i.**

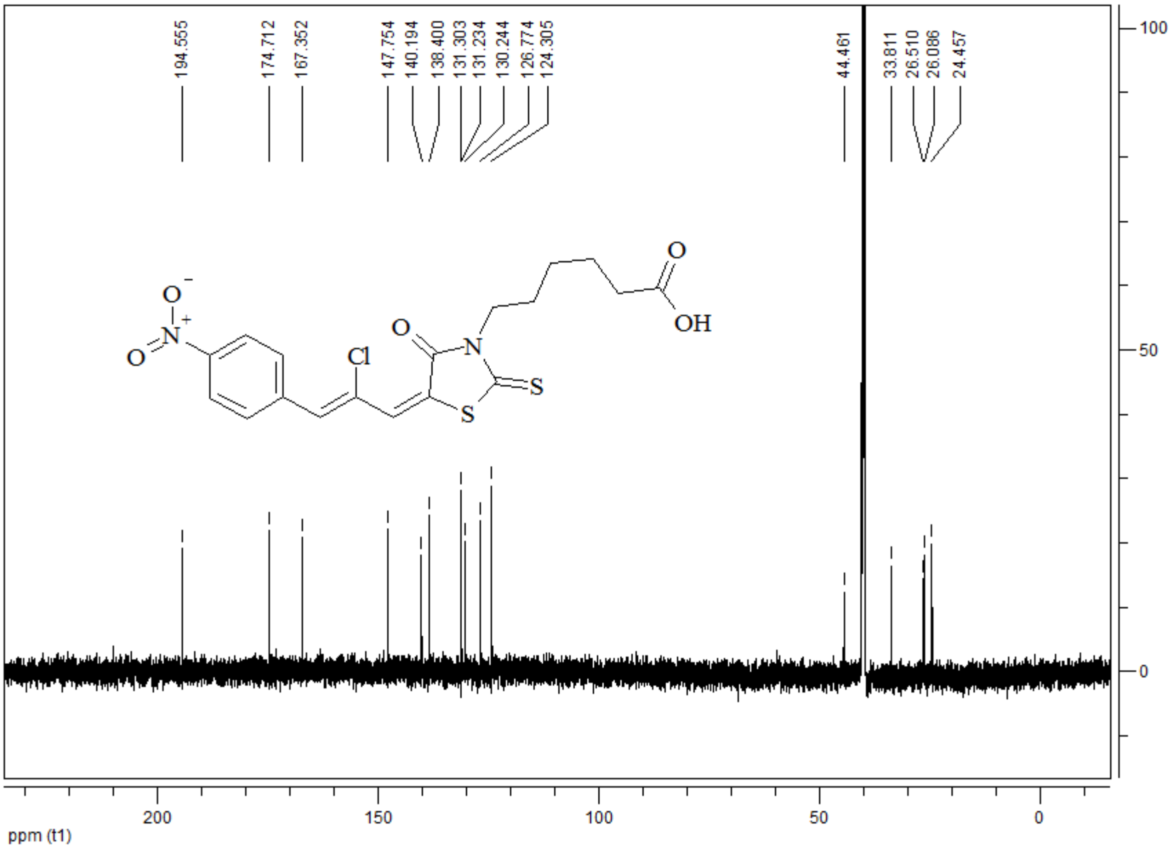

Figure S14. ^{13}C NMR spectrum **2i.**

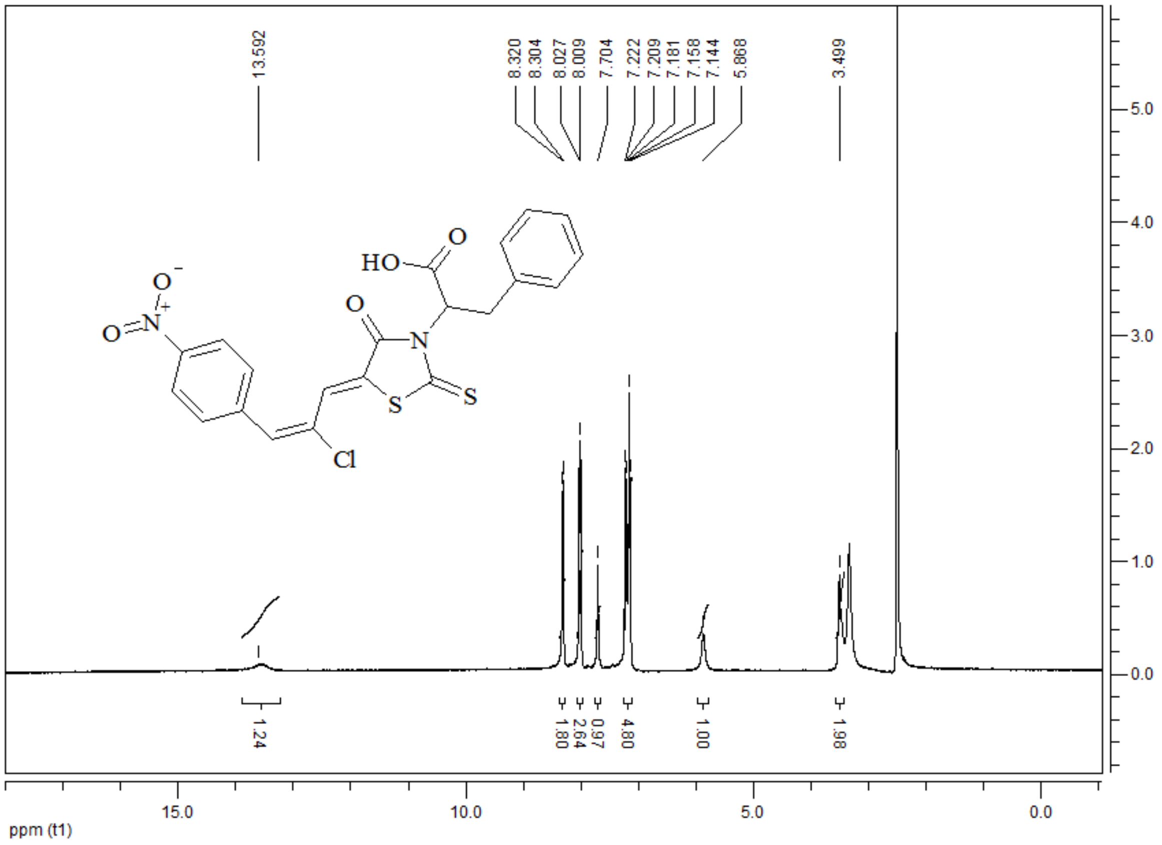

Figure S15. ^{1}H NMR spectrum **2j.**

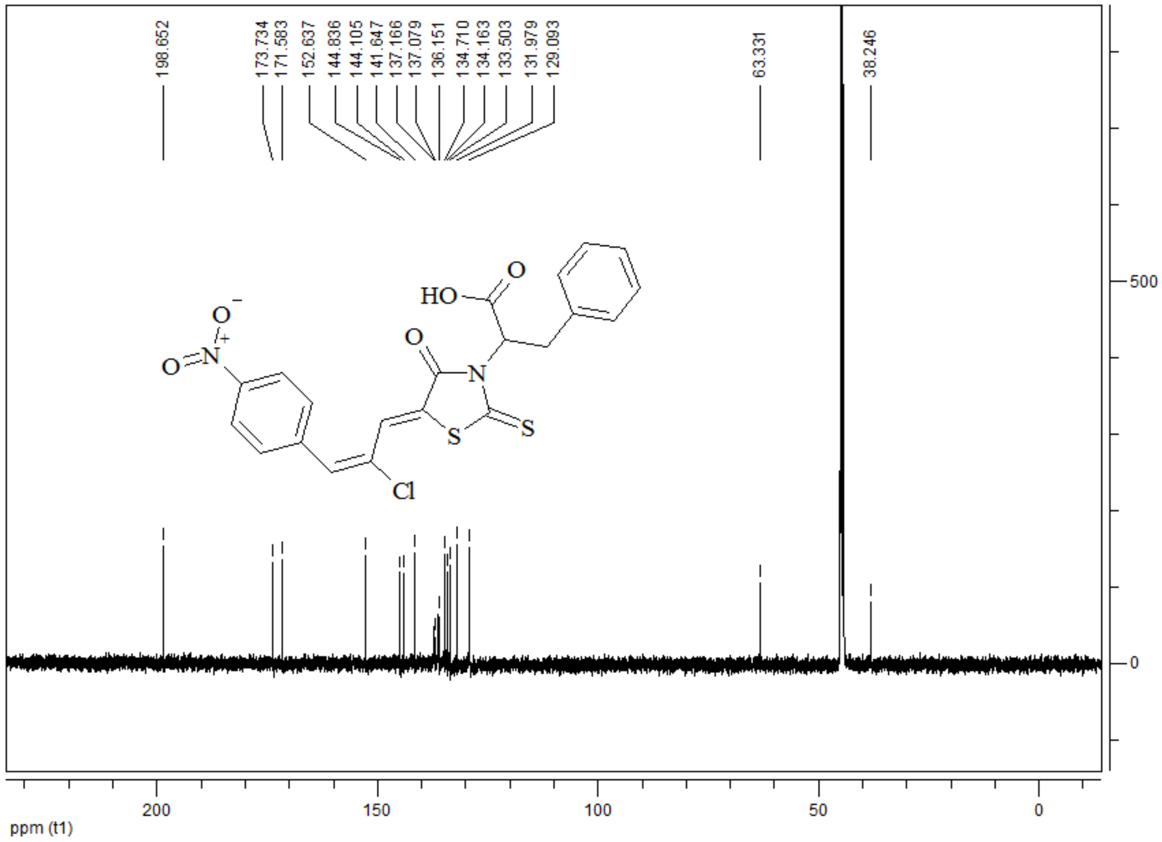

Figure S14. ^{13}C NMR spectrum **2j.**

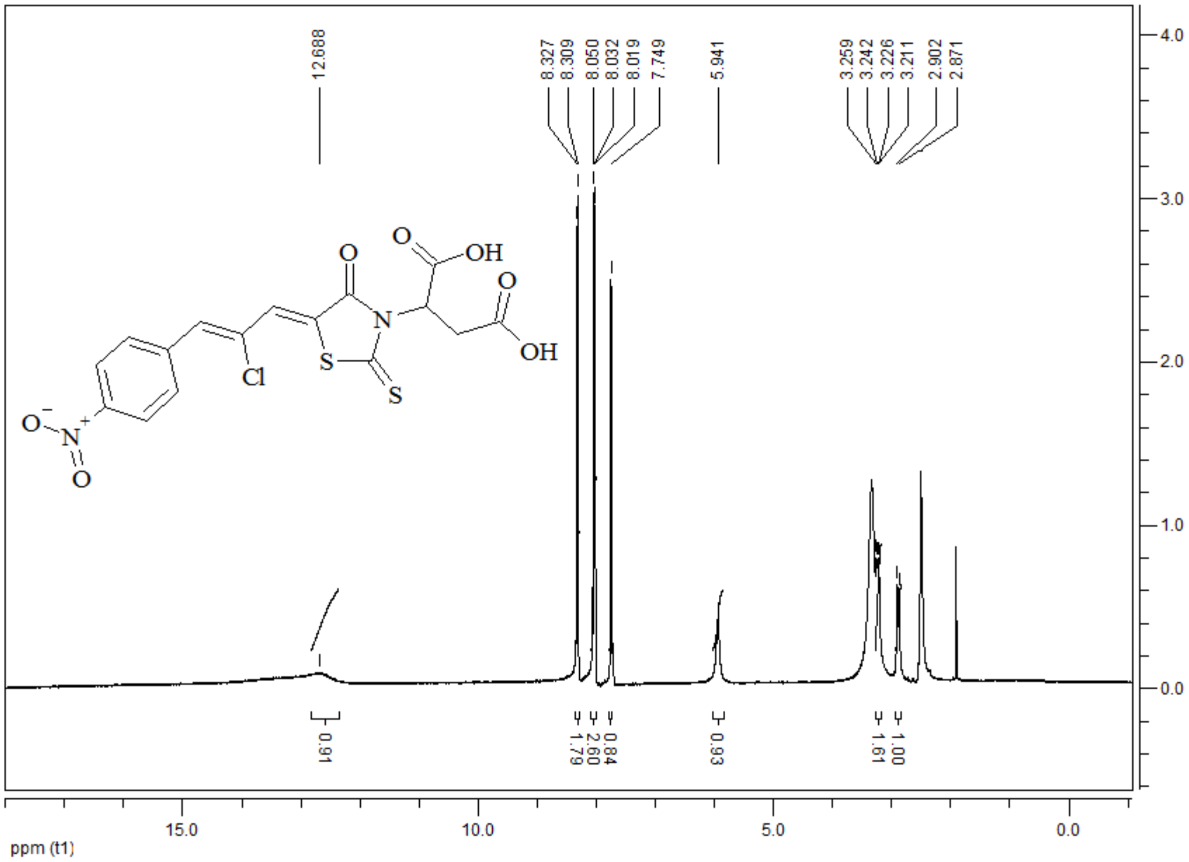

Figure S15. ^{1}H NMR spectrum **2k.**

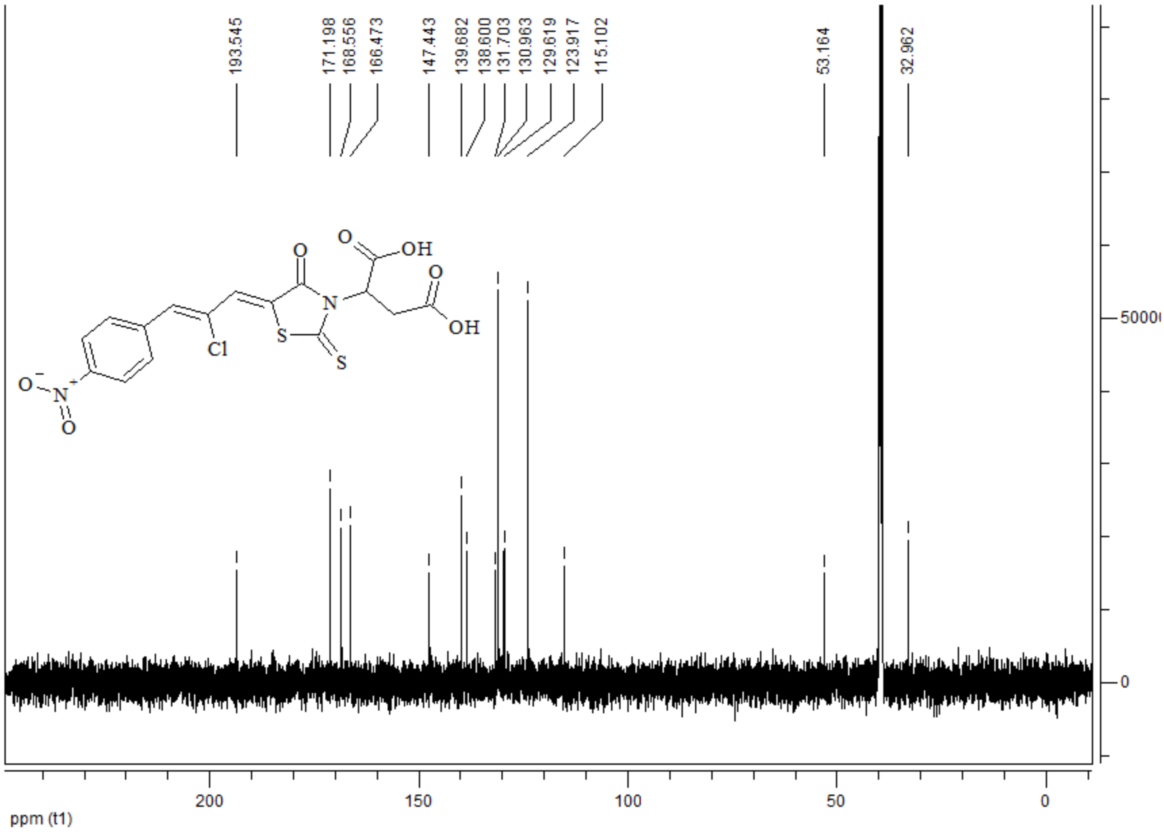

Figure S14. ^{13}C NMR spectrum **2k.**

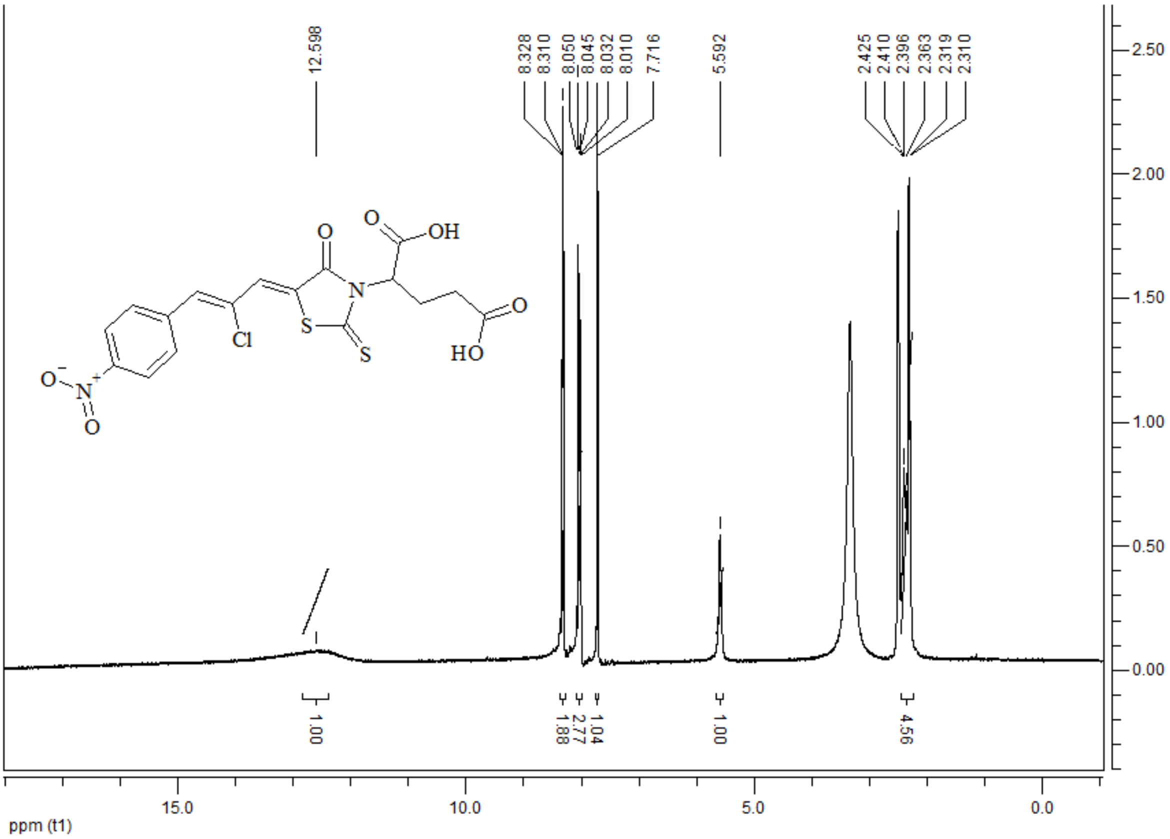

Figure S15. ^{1}H NMR spectrum **2l.**

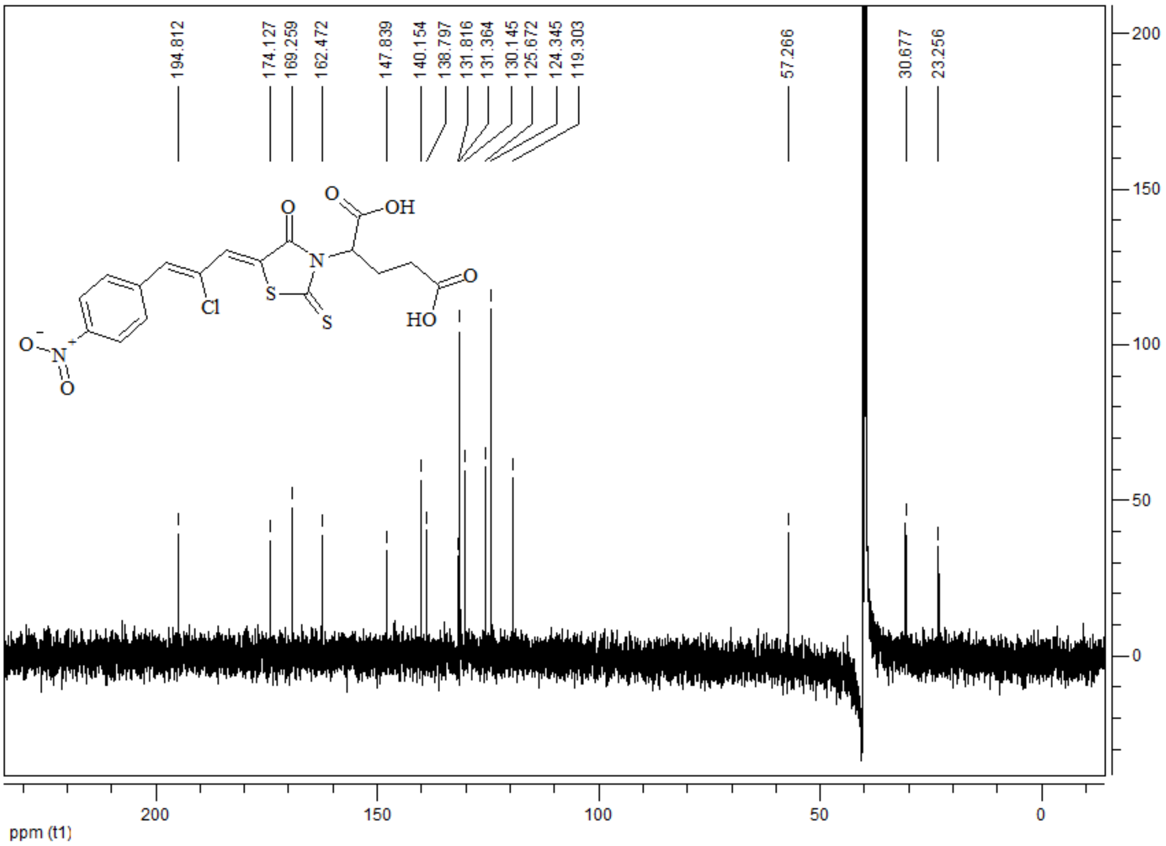

Figure S16. ^{13}C NMR spectrum **2l.**
